# Simplified Preparation of BaAl_2_O_4−*y*_e^−^_*y*_/C Oxy‐Electrides Using Pechini Approach for Ammonia Synthesis

**DOI:** 10.1002/cssc.202500682

**Published:** 2025-06-13

**Authors:** Aissam Addou, Amanda Sfeir, Maya Marinova, Hervé Vezin, Jean‐Philippe Dacquin, Sébastien Royer, Said Laassiri

**Affiliations:** ^1^ Chemical & Biochemical Sciences Green Process Engineering (CBS) Mohammed VI Polytechnic University Benguerir 43150 Morocco; ^2^ Université de Lille CNRS ENSCL Centrale Lille Univ. Artois UMR 8181‐UCCS‐Unité de Catalyse et de Chimie du Solide Lille F‐59000 France; ^3^ Université de Lille CNRS INRA Centrale Lille Université Artois FR 2638–IMEC–Institut Michel‐Eugène Chevreul Lille 59000 France; ^4^ Laboratoire de Spectroscopie pour Les Interactions La Réactivité et L’Environnement Université de Lille UMRCNRS 8516‐LASIRE Lille 59000 France; ^5^ Université du Littoral Côte d’Opale UCEIV UR 4492 MREI‐2 Dunkerque 59140 France

**Keywords:** electride composites, green ammonia, Haber–Bosch, nanoparticles, Ru

## Abstract

Electride‐based materials are among the most active catalysts for ammonia synthesis, with a strong potential for application in the development of green ammonia synthesis; however, their synthesis often requires complicated reaction conditions limiting their scalability. Herein, a straightforward and scalable synthesis method for a BaAl_2_O_4−*y*_e^−^_*y*_/C oxy‐electride composite via carboxylic acid complexation (pseudo‐Pechini) followed by carbothermal reduction is presented. The resulting composite exhibits significant surface area, of ≈41 m^2^ g^−1^, which is considerably higher than the electrides prepared by solid‐state synthesis. Upon loading with ruthenium, the BaAl_2_O_4−*y*_e^−^_*y*_/C composite demonstrates excellent catalytic activity for ammonia synthesis, achieving rates of 3090 μmol g^−1^_catal*y*st_ h^−1^ with 0.6Ru/BaAl_2_O_4−*y*_e^−^_*y*_/C, and 3737 μmol g^−1^_catal*y*st_ h^−1^ with 1.1Ru/BaAl_2_O_4−*y*_e^−^_*y*_/C at mild reaction conditions (400 °C and 1 bar). Comprehensive characterization through X‐ray diffraction, Raman spectroscopy, scanning electron microscopy, and scanning transmission electron microscopy‐energy dispersive X‐ray spectroscopy confirms the textural and structural properties of the catalyst, while iodometric titration method and electron paramagnetic resonance analysis confirm the electride‐like nature of the resulting composite. The results reported in this work highlight a straightforward approach for the design of electride‐based composite material displaying superior catalytic properties for green ammonia synthesis.

## Graphical abstract

This study presents a scalable method to produce a BaAl_2_O_4−*y*_e^−^y/C oxy‐electride composite with appropriate textural, structural, and electronic properties. The resulting catalyst demonstrates excellent catalytic performance at mild conditions. Characterization confirms its electride nature, offering a promising route for green ammonia synthesis.

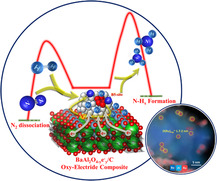

## 1 Introduction

Ammonia (NH_3_), produced at >150 Mt year^−1^, is pivotal for sustaining the world population through providing a straightforward route to nitrogen‐based fertilizers.^[^ ^1^ ^]^ Furthermore, ammonia displays appropriate physical–chemical properties to be considered as key molecule for energy applications, including utilization as fuel in combustion engines, in fuel cells, and as hydrogen carrier for mid‐ and long‐term energy storage.^[^ ^2^, ^3^, ^4^ ^]^ The industrial production of ammonia is exclusively operated by the Haber–Bosch (H–B) process, in which highly purified hydrogen (H_2_), derived from fossil fuels, combines with nitrogen (N_2_) over a doubly promoted iron‐based catalyst.^[^ ^5^, ^6^ ^]^ However, significant CO_2_ is released from ammonia production plants, ≈1.5 tons of CO_2_ per ton of NH_3_, making the current process not sustainable.

Extensive research is dedicated today to replace gray H_2_ by green H_2_ (from water electrolysis powered by renewable energy).^[^ ^7^ ^]^ However, fueling the H–B process by green H_2_ presents significant challenges. The process is optimized for continuous operation and the reaction is conducted at high pressures (150–250 bar) and moderate temperatures (400–500 °C) to obtain an industrially acceptable ammonia yield up to 18 vol%.^[^ ^8^, ^9^ ^]^ Thus, developing novel and agile ammonia production process capable of operating at low pressure (≈50 bar), aligned with pressure delivered by the electrolyze, while coping with intermittent condition is a paramount step for decarbonizing the ammonia industry. By operating under mild conditions, the thermodynamically achievable NH_3_ yield remains comparable to traditional H–B condition.

Shifting from the severe reaction conditions of the H–B process to mild conditions relies on the development of new catalysts, that remain highly active at lower temperatures. Ru drives significant interest due to its high catalytic activity stemming from its appropriate nitrogen adsorption‐activation abilities.^[^ ^9^ ^]^ Despite not industrially running today, Ru‐graphite catalyst was proposed for the Kellogg Advanced Ammonia Process (KAAP), a process operated at lower pressure than the H–B process.^[^ ^10^ ^]^ Unfortunately, Ru suffers from H_2_ poisoning at high pressure or in rich H_2_ limiting its applications. Over the past few decades, strategies were developed to ameliorate Ru performance. Particularly, supporting Ru on functional supports such as C12A7:e^−^, BaAl_2_O_4−*x*_H_*y*_, BaYO_2_H, BaScO_2_H, BaTiO_3−*x*_H_*x*_, BaCeO_3–*x*_N_*y*_H_z_, and Ba‐Ca(NH_2_)_2_, Ca_2_NH, BaO_*x*_N_*y*_:e^−^_*z*_, Ca_2_N, LaScSi intermetallic leads to remarkable performances.^[^ ^11^, ^12^, ^13^, ^14^, ^15^, ^16^, ^17^, ^18^, ^19^, ^20^ ^]^ The utilization of inorganic electride has been reported to be particularly efficient in promoting Ru activity, lowering the apparent activation energy (*E*_a_) of the reaction (e.g., Ru/C12A7:e^−^, Ru/BaAl_2_O_4−*x*_H_*y*_) to 50–70 kJ mol^−1^ and allowing to work at lower temperatures (<400 °C). The ability of electride to host anionic electrons in their interstitial cage sites favors electron donation to Ru‐B5 active sites, and by consequence enhances the back‐donation of electrons to N_2_. In the particular case of electrides, the rate‐determining steps is no longer the cleavage of the strong N≡N bonds (Dissociation energy 945 kJ mol^−1^),^[^ ^21^ ^]^ but the subsequent hydrogenation of nitrogen‐activated species.^[^ ^22^ ^]^

However, electride‐based materials synthesis is often achieved through solid‐state reactions requiring high temperatures (1300–1600 °C) under controlled atmosphere (H_2_, Ar, CO/CO_2_ mixture) or vacuum.^[^ ^23^, ^24^, ^25^, ^26^ ^]^ Additionally, difficult‐to‐handle precursors such as BaH_2_, CaH_2_, Ca, Al, and Ti (all prone to decomposition/oxidation under ambient conditions) are used for the synthesis.^[^ ^11^, ^12^, ^16^, ^18^, ^27^, ^28^, ^29^, ^30^ ^]^ One into the other, synthesis scalability becomes complex and electrides, often microcrystalline solids, displaying low surface area which render Ru dispersion and stabilization particularly problematic. Therefore, the development of syntheses from available, low cost, stable chemicals is worthy of interest in view of industrial scale‐up.

In this work, we propose to use a pseudo‐Pechini approach, combined with carbothermal reduction to produce BaAl_2_O_4−*y*_e^−^_*y*_/C oxy‐electride. BaAl_2_O_4−*x*_H_*y*_ electride has been recently reported as suitable support for Co or Ru (Ru (2 wt%) /BaAl_2_O_4–*x*_H_*y*_ produces 13,304 μmol g_catal*y*st_^−1^ h^−1^ at weight hourly space velocity (WHSV) 36,000 mL g_catal*y*st_^−1^ h^−1^, 340 °C and 9 bar).^[^ ^11^ ^]^ The structure possesses interstitial cage sites which can accommodates (nucleus‐free) anionic electrons, and displays very low surface work function (1.7–2.6 eV).^[^ ^11^ ^]^ Extensive mechanistic studies by Hosono *et al.* have shown that BaAl_2_O_4−*x*_H_*y*_ surfaces can achieved exceptionally low work function, primarily due to “nucleus‐free” anionic electrons, localized at hydrogen vacancies (V_H_) in close proximity to Ba on the surface.^[^ ^11^ ^]^ These electrons are easily donated to the supported metal (e.g., Co, Ru), thereby lowering the barrier for direct N_2_ dissociation which is widely regarded as the rate‐determining step in ammonia synthesis.^[^ ^22^, ^31^, ^32^, ^33^ ^]^ In addition, the presence of lattice H^−^ ions accelerates the hydrogenation of the surface‐bound nitrogen intermediates and serves as a reversible hydrogen reservoir, further enhancing the overall catalytic performance.^[^ ^11^, ^33^, ^34^ ^]^ BaAl_2_O_4−*x*_H_*y*_ was synthesized by the solid‐state reaction between γ‐Al_2_O_3_, BaH_2_, and BaCO_3_. The reaction was conducted under H_2_ gas flow, at 800 °C for 20 h, resulting in a low surface area (≈5 m^2^ g^−1^).

In this work, electride‐based composite was prepared using inexpensive and stable nitrates solubilized in water, complexed with citric acid and ethylene glycol, both acting as carbon source for the final composite. The carbothermal reduction step is conducted at 1400 °C under N_2_, leading to the formation of BaAl_2_O_4−*y*_e^−^_*y*_/C oxy‐electrides composites with high surface areas (≈41 m^2^ g^−1^). When compared to classical pathways for mixed‐oxide and electride synthesis, the modified Pechini method offers several advantages in both cost and scalability. In contrast to alkoxide‐based sol‐gel approaches, which often involve more expensive alkoxide precursors, or solid‐state ceramic routes relying on air/moisture sensitives metal hydrides, the modified Pechini method uses readily available, lower‐cost reagents (e.g., metal nitrates, citric acid, ethylene glycol) under atmospheric conditions. The process promotes molecular‐level mixing of the mixed oxide components, yielding highly homogeneous gels with controlled stoichiometry. Furthermore, every step of the present approach is scalable using conventional chemical engineering principles, making it industrial‐friendly compared to methods requiring vacuum equipment, gloveboxes, or the use of hazardous precursors.

Upon low content Ru impregnation (<1 wt%), composites achieve excellent performances in NH_3_ synthesis: activity of 0.6Ru/BaAl_2_O_4−*y*_e^−^_*y*_/C is 3090 μmol g_catal*y*st_^−1^ h^−1^ at WHSV 36,000 mL g_catal*y*st_^−1^ h^−1^, 400 °C and 1 bar. Apparent *E*_*a*_ measured fall within the range (50–80 kJ mol^−^^1^) reported for active catalysts from the literature (Ru/C12A7:e^−^, Ru/BaO_*x*_N_*y*_:e^−^_z_, Ru/BaAl_2_O_4−*x*_Hy, BaScO_2_H, BaYO_2_H, Ru/BaTiO_2.5_H_0.5_).^[^ ^11^, ^12^, ^19^, ^20^, ^34^ ^]^

## 2 Results

### 2.1 Structural Characterization of BaAl_2_O_4−*y*_e^−^_*y*_/C

**Figure 1:**
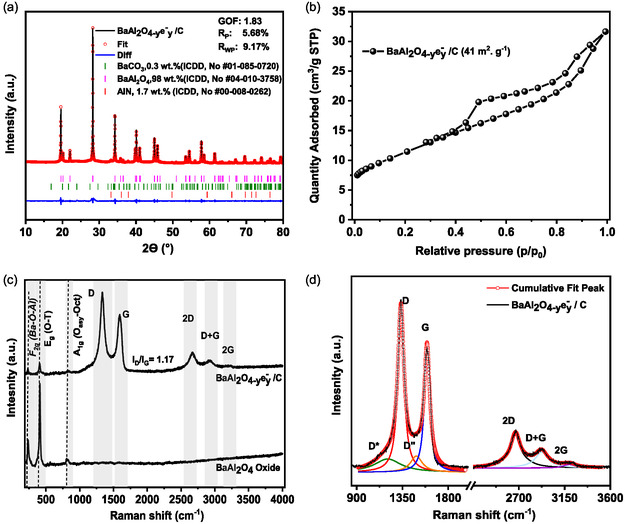
Structural and textural characterizations. a) XRD pattern of BaAl_2_O_4−*y*_e^−^_*y*_/C (after carbothermal reduction step). b) Nitrogen adsorption–desorption isotherm of BaAl_2_O_4−*y*_e^−^_*y*_/C. c) Raman spectra of BaAl_2_O_4−*y*_e^−^_*y*_/C and BaAl_2_O_4_ oxide. d) Raman spectra deconvolution of BaAl_2_O_4−*y*_e^−^_*y*_/C.

BaAl_2_O_4−*y*_e^−^_*y*_/C oxy‐electride composite was produced by a straightforward approach involving a first complexation of readily available precursors, followed by a carbothermal reduction step at 1400 °C conducted under N_2_. X‐ray diffraction (XRD) pattern recorded after carbothermal reduction is presented in **Figure** **1**a. Pattern exhibited sharp and intense reflections indicative of a well‐crystallized material with a large crystal domain size. The main diffraction peaks are all associated with BaAl_2_O_4_ structure (ICDD, PDF #04‐010‐3758) of hexagonal structure (space group P6_3_22(182)). Minor impurities are detected: BaCO_3_ (ICDD, PDF #01‐085‐0720) at 2*θ* = 24°, and AlN (ICDD, PDF #00‐008‐0262) at 2*θ* = 33, 38°. The results of Rietveld refinement confirm the stabilization of BaAl_2_O_4_ phase (≈98 wt%) after the carbothermal reduction step. Oxide BaAl_2_O_4_, obtained by calcination at 1000 °C, presents similar pattern to the BaAl_2_O_4−*y*_e^−^_*y*_/C composite (Figure S1, Supporting Information). Lattice parameters from Rietveld refinement for both samples are summarized in Table S1, Supporting Information.

Residual carbon content in BaAl_2_O_4−*y*_e^−^_*y*_/C, formed during carbothermal step, was determined by thermogravimetric analysis (TGA) (**Table** **1**; Figure S2, Supporting Information). At low temperature (<200 °C), only a small weight loss (1.5 wt%) is associated to water removal which hints on the hydrophobic nature of the material surface. Figure S2, Supporting Information, reveals a weight loss of ≈20 wt% between 250 and 700 °C. Differential thermal analysis (DTA) (Figure S2, Supporting Information) shows that carbon oxidation process is dominated by two distinct thermal events, likely associated with the combustion of carbon having varying stability: amorphous carbon or partially graphitized carbon combustion, followed by stable graphitized carbon skeleton oxidation.^[^ ^35^ ^]^

**Table 1:** Properties of BaAl_2_O_4−*y*_e^−^_*y*_/C composite and BaAl_2_O_4_ oxide.

Sample	Surface areaa) [m^2^ g^−1^]	Carbon contentb) [wt%]	Nec) [10^21^ cm^−3^]
BaAl_2_O_4_	<2	n.d.	0.00
BaAl_2_O_4−*y*_e^−^_*y*_/C	41	20.3 ± 0.1	5.7 ± 0.5

a) *S*_BET_, surface area obtained applying the BET method;

b) carbon content estimated from TGA analysis;

c) Ne electron density determined by iodometry titration method. n.d. stands for “not determined”.

BaAl_2_O_4_ and BaAl_2_O_4−*y*_e^−^_*y*_/C underwent additional characterization by Raman spectroscopy (Figure 1c,d). The Raman spectra of BaAl_2_O_4_ show the presence of peaks at 236 and 410 cm^−1^ corresponding to F_2g_ and E_g_ vibrational modes of BaAl_2_O_4_ structure.^[^ ^36^ ^]^ Although weaker in intensity, similar F_2g_ and E_g_ vibrational modes were observed for BaAl_2_O_4−*y*_e^−^_*y*_/C confirming that BaAl_2_O_4_ phase remains after the carbothermal reduction step, which is consistent with XRD results. Furthermore, Raman spectra of BaAl_2_O_4−*y*_e^−^_*y*_/C evidenced two signals: D (defect) band at 1332 cm^−1^ and G band (graphitic) at 1580 cm^−1^, Figure 1c,d. The G peak is related to the first‐order E_2g_ phonon mode of graphite and the in‐plane stretching of the C=C bonds, while the D peak is assigned to the A_1g_ breathing mode at the Brillouin zone boundary K, which corresponds to the defects in the graphene plane.^[^ ^37^ ^]^ The G peak position is in line with s*p* ^2^ dominated carbonaceous materials (graphite, carbons black, activated carbons^[^ ^38^, ^39^ ^]^). The I_D_/I_G_ ratio is measured to be ≈1.17. This value indicates the degree of graphitization, which is associated in the literature with poor crystallinity.^[^ ^40^ ^]^ Second‐order bands (2D, D+G, and 2G) are observed in the range of 2250 to 3200 cm^−1^. The 2D band, ≈2664 cm^−1^, is generally visible in the Raman spectra of graphene or s*p* ^2^ carbon. The 2G band, at 3160 cm^−1^, is attributed to the overtone of the G band, while the D+G band, at 2912 cm^−1^, is the combined overtone of D and G bands. Raman spectra deconvolution, Figure 1d, evidences the presence of D* band at ≈1205 cm^−1^, assigned to disordered graphitic lattice provided by s*p* ^2^–s*p* ^3^ bonds at carbon network edges. I_D*_/I_G_ ratio of ≈0.11 is measured, which closely aligned with ratio of reduced graphene oxide,^[^ ^41^ ^]^ that confirms the efficiency of the carbothermal reduction step in eliminating terminal oxygen‐containing groups. Additionally, the presence of the D″ band around 1486 cm^−1^ is attributed to the presence of the amorphous phase of carbon.^[^ ^42^ ^]^ Furthermore, no Raman bands were detected in the 2100–2200 cm^−1^ spectral region, where C≡N stretching vibrations typically appear,^[^ ^43^, ^44^ ^]^ confirming the absence of nitrogen‐doped carbon species in these conditions.

To sum up, Raman spectroscopy shows that during the carbothermal reduction step, organic matter is subject to graphitization leading to the formation of poorly crystalline, defective s*p* ^2^ carbon with the presence of disorder related to the carbon s*p* ^2^–s*p* ^3^ in the lattice of the graphitized carbon.

Textural properties of BaAl_2_O_4−*y*_e^−^_*y*_/C are obtained by N_2_ physisorption analysis (Figure 1b, Table 1). The composite exhibited a combined characteristics of type‐II (poorly porous) and type IV isotherm profile. A hysteresis loop that resembles H4 is observed. H4 hysteresis is often observed with micro‐mesoporous carbons which is coherent with the retained carbon phase in the final catalyst. The surface area, Table 1, is ≈41 m^2^ g^−1^ for BaAl_2_O_4−*y*_e^−^_*y*_/C. In contrast, BaAl_2_O_4_ obtained after the calcination step displays a very limited surface area, <2 m^2^ g^−1^. Therefore, the surface area of the oxy‐electride composite is likely attributed to the presence of graphitized carbon formed during the carbothermal reduction process, inducing porosity and reducing inorganic phase sintering.

### 2.2 Morphological Characterization of BaAl_2_O_4−*y*_e^−^_*y*_/C

The morphology of the BaAl_2_O_4−*y*_e^−^_*y*_/C was studied using SEM, Figure S3, Supporting Information. Formation of particle aggregates, which appeared as randomly shaped particles of microscale dimensions. As illustrated by XRD pattern, most of the aggregates contain all elements in which BaAl_2_O_4−*y*_e^−^_*y*_ phase and carbon are well intertwined (Figure S3, Supporting Information). Macroscopic elemental distribution is homogeneous among the aggregates except on isolated grains as observed in Figure S3, Supporting Information, where some enrichment of Al and N are observed.

**Figure 2:**
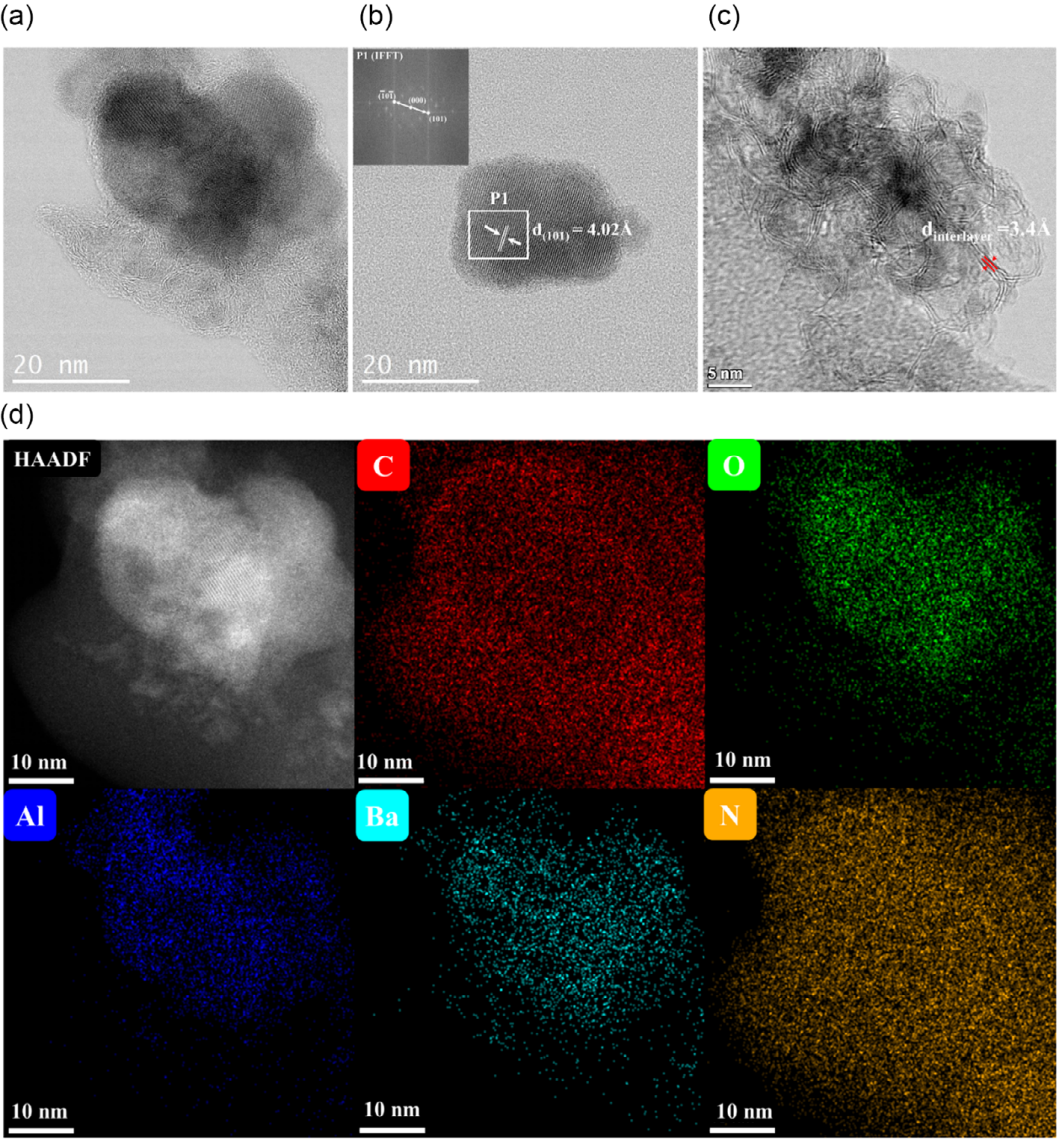
a–c) BF‐STEM image of BaAl_2_O_4−*y*_e^−^_*y*_/C. d) Representative HAADF‐STEM images coupled with EDS elemental mapping of BaAl_2_O_4−*y*_e^−^_*y*_ /C.

To gain a more precise understanding of the morphology of BaAl_2_O_4−*y*_e_*y*_/C composite and the distribution of its constituent elements, transmission electron microscopy (TEM) and scanning transmission electron microscopy (STEM), coupled with energy dispersive X‐ray spectroscopy (EDS) elemental mapping analyses, were performed (**Figure** **2** and Figure S4–S6, Supporting Information). The presence of crystallized BaAl_2_O_4_ is confirmed in the high‐angle annular dark‐field (HAADF)‐STEM and TEM images. Interplanar distances of 4.01 and 3.16 Å, corresponding to (101) and (102) crystallographic planes of the hexagonal BaAl_2_O_4_ structure (ICDD, PDF #01‐085‐0720) are observed (Figure 2b; Figure S5, Supporting Information). In some cases, phases of different composition surrounding BaAl_2_O_4_ particles are observed, EDS elemental mapping suggesting BaCO_3_ or BaO impurity formation (Figure S6, Supporting Information). Bright‐field (BF)‐STEM images (Figure 2c; Figure S4, Supporting Information) revealed the graphitized carbon exists either in interaction with BaAl_2_O_4_ or as free filamentous structures forming mono‐ or multilayers. Such distribution is observed consistently across other regions. The measured interlayer spacing between carbon layer, 0.34 ± 0.012 nm, remains close to the values reported for interlayer spacing in carbon materials such as reduced graphene oxide (0.357 nm),^[^ ^45^ ^]^ expanded graphite (0.37 nm),^[^ ^46^ ^]^ filamentous carbon (0.3342 nm),^[^ ^47^ ^]^ and multiwalled carbon nanotubes (0.32–0.35 nm).^[^ ^48^ ^]^ As already suggested by SEM‐EDS analysis, nitrogen incorporation in the material is confirmed by STEM‐EDS as well which is consistent with AlN formation under carbothermal reduction under N_2_ atmosphere.^[^ ^49^, ^50^, ^51^ ^]^

### 2.3 Electride Nature of the BaAl_2_O_4−*y*_e^−^_*y*_/C

Electrons trapping in BaAl_2_O_4−*y*_e^−^_*y*_/C is evaluated using iodometric titration and electron paramagnetic resonance (EPR) spectroscopy. From iodometric titration, electrons trapped in the sample were measured to be ≈5.72 × 10^21^ cm^−3^ for the BaAl_2_O_4−*y*_e^−^_*y*_/C, while no reductive species (e^−^) were measured for BaAl_2_O_4_ type oxide (Table 1). Structural defects within the BaAl_2_O_4_ lattice give rise to the origin of the measured electron density. The value measured here is superior to value reported for BaAl_2_O_4−*x*_H_*y*_ (2.0 × 10^21^ cm^−1^) prepared by solid‐state reaction between γ‐Al_2_O_3_ and BaH_2_ in the presence of BaCO_3_ under H_2_ flow.^[^ ^11^ ^]^ The highest electronic density in BaAl_2_O_4−*y*_e^−^_*y*_/C might originate from the presence of structural defects in graphitized carbon which can also act as trapping centers for electrons, leading to increased reduction of I_2_ into I^−^ ions.^[^ ^52^ ^]^

Furthermore, the EPR spectrum shown in Figure S7, Supporting Information, clearly indicates that the BaAl_2_O_4−*y*_e^−^_*y*_/C composite support contains localized electrons, as evidenced by its isotropic Lorentzian peak shape a signal already reported for reduced BaAl_2_O_4_ (electron‐trapped center in oxygen vacancies).^[^ ^53^, ^54^ ^]^ The g‐factor of the BaAl_2_O_4−*y*_e^−^_*y*_/C composite support was measured to be ≈2.006. Although in the literature, graphitized carbon‐based materials with defects exhibit as well an EPR signals with g‐factors centered around ≈2.002.^[^ ^55^ ^]^ While the EPR spectrum of BaAl_2_O_4−*y*_e^−^_*y*_/C, does not exhibit multiple distinct components, the resulting over‐all signal might originate from the contribution of both partially reduced BaAl_2_O_4_ and the graphitized carbon. In contrast, no signal was observed for the oxide support.

The reducibility of BaAl_2_O_4−*y*_e^−^_*y*_/C is studied by temperature‐programmed reduction (TPR) under 5 vol% H_2_ in Ar, and the results compared to the BaAl_2_O_4_ oxide counterpart. As presented in Figure S8, Supporting Information, the reduction profile of BaAl_2_O_4_ is characterized by a main reduction peak at 800 °C (associated to a small hydrogen consumption at 894 °C). These features are consistent with the literature, where the reduction process is associated with the creation of oxygen vacancies in BaAl_2_O_4_ lattice.^[^ ^53^ ^]^ At the opposite, TPR profile of BaAl_2_O_4−*y*_e^−^_*y*_/C exhibits a negligible hydrogen consumption between 400 and 800 °C, indicating that the carbothermal step resulted in the reduction of BaAl_2_O_4−*y*_e^−^_*y*_/C, and its stabilization at the reduced state.

### 2.4 Characterization of Ru/BaAl_2_O_4−*y*_e^−^_*y*_/C

#### 2.4.1 Properties of Ru/BaAl_2_O_4−y_e^−^_y_/C

**Figure 3:**
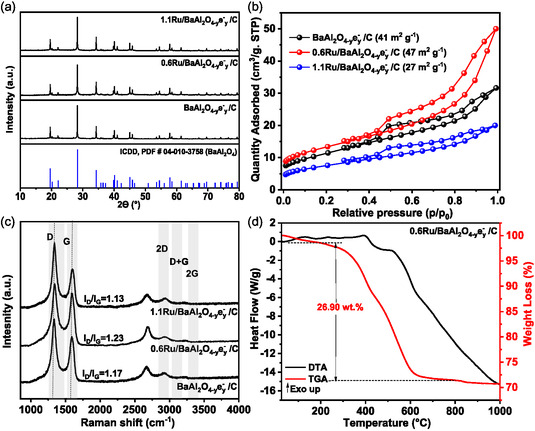
0.6Ru/BaAl_2_O_4−*y*_e^−^_*y*_/C and 1.1Ru/BaAl_2_O_4−*y*_e^−^_*y*_/C characterization. a) XRD pattern. b) N_2_ physisorption isotherms. c) Raman spectra. d) TGA‐DTA profile under air obtained for 0.6Ru/ BaAl_2_O_4−*y*_e^−^_*y*_/C.

Ru/BaAl_2_O_4−*y*_e^−^_*y*_:C catalysts with different Ru loading were prepared by impregnation method. XRD of 0.6Ru/BaAl_2_O_4−*y*_e^−^_*y*_/C and 1.1Ru/BaAl_2_O_4−*y*_e^−^_*y*_/C are presented in **Figure** **3**a. Only diffractions peaks of BaAl_2_O_4_ phase (ICDD, PDF #04‐010‐3758) are observed. The absence of reflection related to Ru in both catalysts can be ascribed of either a good dispersion of the active phase within the support or a limited crystal size (<2 nm) for the stabilized nanoparticles. After Ru impregnation and thermal treatment, 0.6Ru/BaAl_2_O_4−*y*_e^−^_*y*_/C and 1.1Ru/BaAl_2_O_4−*y*_e^−^_*y*_/C show type IV isotherm (Figure 3b), similar to BaAl_2_O_4−*y*_e^−^_*y*_/C. The surface area only varies slightly: 47 m^2^ g^−1^ for 0.6Ru/BaAl_2_O_4−*y*_e^−^_*y*_/C/ and 24 m^2^ g^−1^ for 1.1Ru/BaAl_2_O_4−*y*_e^−^_*y*_/C (vs 41 m^2^ g^−1^ for BaAl_2_O_4−*y*_e^−^_*y*_/C).

Raman spectra of 0.6Ru/BaAl_2_O_4−*y*_e^−^_*y*_/C and 1.1Ru/BaAl_2_O_4−*y*_e^−^_*y*_/C catalysts are presented in Figure 3c. As observed in the support BaAl_2_O_4−*y*_e^−^_*y*_/C, the Raman spectra of the catalysts were dominated by the presence of the D (defect) band at 1332 cm^−1^ and the G band (graphitic) at 1580 cm^−1^. The I_D_/I_G_ ratio was found to be ≈1.23 for 0.6Ru/BaAl_2_O_4−*y*_e^−^_*y*_/C and ≈1.13 for 1.1Ru/BaAl_2_O_4−*y*_e^−^_*y*_/C. These results indicate that no notable changes in the nature of the graphitized carbon are observed after the impregnation and decomposition of ruthenium carbonyl steps. Furthermore, the quantity of carbon in both catalysts (Figure 3d; Figure S9, Supporting Information) remains comparable than the one from BaAl_2_O_4−*y*_e^−^_*y*_/C (0.6Ru/BaAl_2_O_4−*y*_e^−^_*y*_/C: 26.9 wt%; 1.1Ru/BaAl_2_O_4−*y*_e^−^_*y*_/C: 20.2 wt%). These results indicate no notable changes on the quantity and nature of the carbon from the composite, after Ru deposition.

**Figure 4:**
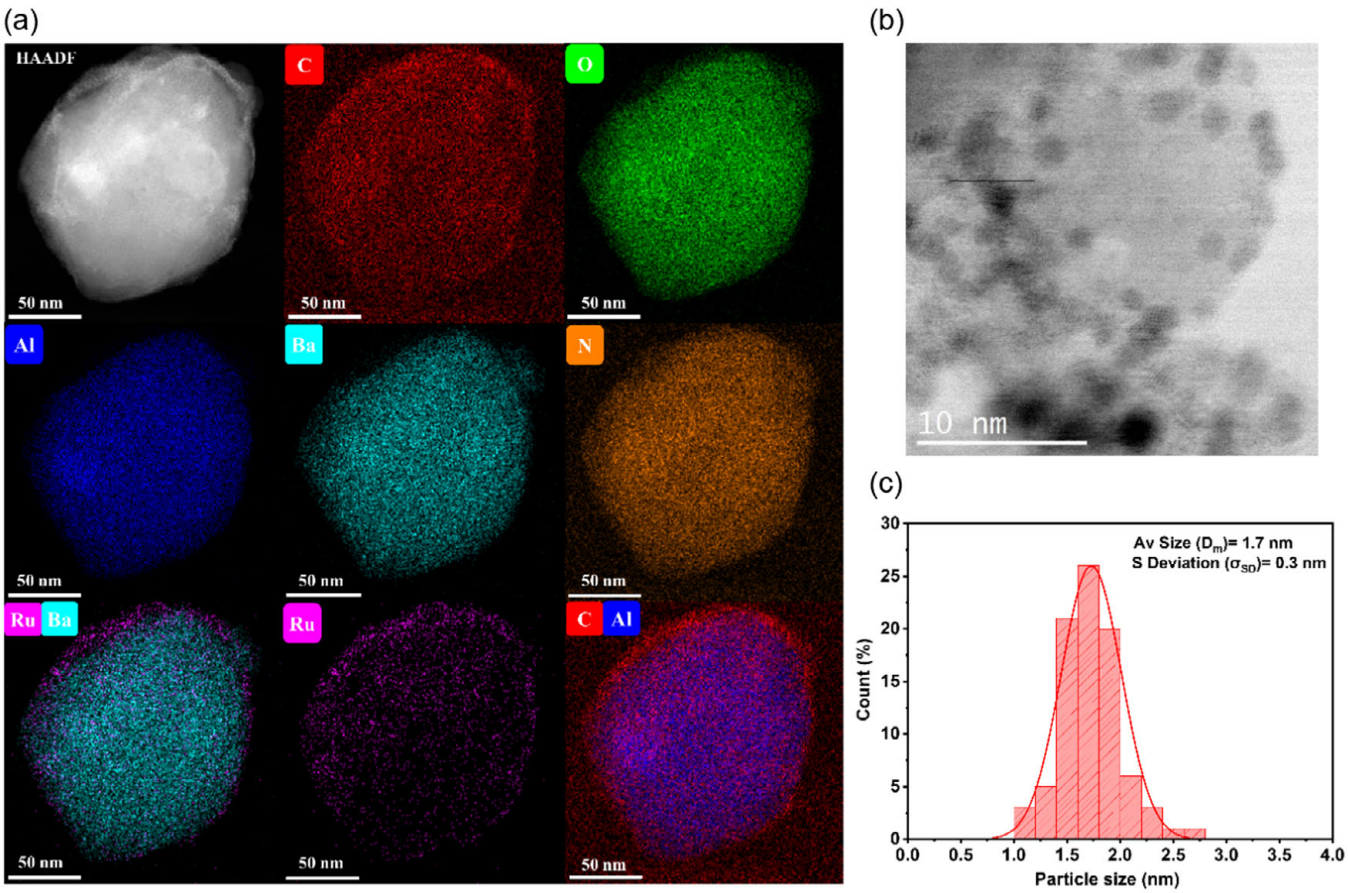
a) HAADF‐STEM image coupled with EDS elemental mapping; b) STEM image; and c) corresponding Ru particle size statistical analysis for catalyst (average of 90 particles counted for each sample).

Ru dispersion was evaluated by (S)TEM (**Figure** **4**; Figure S10–S12, Supporting Information). Analysis reveals that Ru nanoparticles in 0.6Ru/BaAl_2_O_4−*y*_e^−^_*y*_/C are well distributed at the surface of BaAl_2_O_4−*y*_e^−^_*y*_/C particles (Figure 4a,b; Figure S10, Supporting Information). Interplanar spacing of Ru lattice (Figure S12a,b, Supporting Information) of 0.198–0.204 nm, corresponding to the (101) plane is measured. BaAl_2_O_4_ lattice fringes are also observed: (110) plane of 0.263 nm, corresponding to the X‐ray diffraction peak at 34.31° for BaAl_2_O_4_. This observation supports that Ru disperses on BaAl_2_O_4−*y*_e^−^_*y*_/C, and not on impurities identified (AlN, BaCO_3_). Statistical analysis shows an average size of 1.7 ± 0.3 nm (Figure 4c). Comparable particle localization and sizes are obtained for 1.1Ru/BaAl_2_O_4−*y*_e^−^_*y*_/C (Figure S11, Supporting Information). The measured sizes are close to the “ideal” Ru particle size (1.8–2 nm) to maximize B5 sites exposure, sites responsible for the dissociation of N‐bonds into dinitrogen atoms via a dissociative mechanism.^[^ ^56^ ^]^

#### 2.4.2 Oxidation States of Ruthenium Over Catalysts

**Figure 5:**
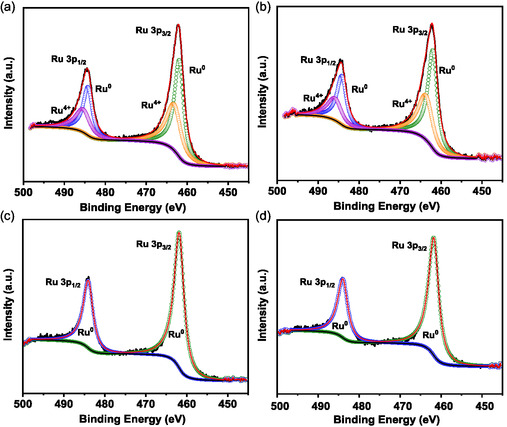
XPS profiles of the Ru 3p region for a) 0.6Ru/BaAl_2_O_4−*y*_e^−^_*y*_/C; b) 1.1Ru/BaAl_2_O_4−*y*_e^−^_*y*_/C; c) 0.6Ru/BaAl_2_O_4−*y*_e^−^_*y*_/C after 2h under ammonia synthesis conditions; and d) 1.1Ru/BaAl_2_O_4−*y*_e^−^_*y*_/C after 2h under ammonia synthesis conditions.

X‐ray photoelectron spectroscopy (XPS) measurements were carried out to gain information on the oxidation state of Ru species. A pretreatment chamber was utilized to investigate the evolution of the oxidation state of Ru under reaction conditions. The results are presented in **Table** **2** and **Figure** **5**.

**Table 2:** Position of Ru 3p peaks of 0.6Ru/BaAl_2_O_4−*y*_e^−^_*y*_/C and 1.1Ru/BaAl_2_O_4−*y*_e^−^_*y*_/C.

	Ru peaks				Surface composition	
	Ru^0^		Ru^4+^		Ru^0^	Ru^4+^
	3*p*_3/2_	3*p*_1/2_	3*p*_3/2_	3*p*_1/2_	[%]	[%]
0.6Ru/BaAl_2_O_4−*y*_e^−^_*y*_/C	461.93	484.13	463.18	485.58	56.2	43.8
0.6Ru/BaAl_2_O_4−*y*_e^−^_*y*_/Ca)	461.82	484.02	–	–	100	–
1.1Ru/BaAl_2_O_4−*y*_e^−^_*y*_/C	461.98	484.18	463.73	485.93	59.3	40.7
1.1Ru/BaAl_2_O_4−*y*_e^−^_*y*_/Ca)	461.75	483.95	–	–	100	–

a) The catalyst was pretreated under reaction conditions (60 mL min^−1^ of 75 vol% H_2_ in N_2_) at 400 °C for 2 h in an environmental XPS chamber prior to analysis.

Upon Ru carbonyl impregnation and subsequent decomposition under N_2_, the high‐resolution Ru 3*p* XPS spectra of 0.6Ru/BaAl_2_O_4−*y*_e^−^_*y*_/C showed the co‐existence of Ru^4+^ and Ru^0^ (Figure 5a). The peaks located at 461.9 and 484.2 eV correspond to Ru 3*p*_3/2_ and Ru 3*p*_1/2_ for metallic Ru, respectively, while the peaks located at 463.2 and 485.4 eV correspond to the Ru^4+^.^[^ ^57^, ^58^ ^]^ Similar Ru species distribution was observed for 1.1Ru/BaAl_2_O_4−*y*_e^−^_*y*_/C catalysts with slightly higher concentration of Ru metallic species (59.3 at%) compared to 0.6Ru/BaAl_2_O_4−*y*_e^−^_*y*_/C (56.2 at %).

After 2 h under ammonia synthesis conditions, at 400 °C, the XPS spectra showed only peaks associated with metallic Ru, indicating the complete reduction of Ru^4+^ to Ru^0^ during the initial hours of the catalytic reaction (Figure 5c,d). This suggests that the reaction environment facilitates the transformation of the remaining oxidized Ru species into the active metallic form.

### 2.5 Catalytic Performances for Ammonia Synthesis

**Figure 6:**
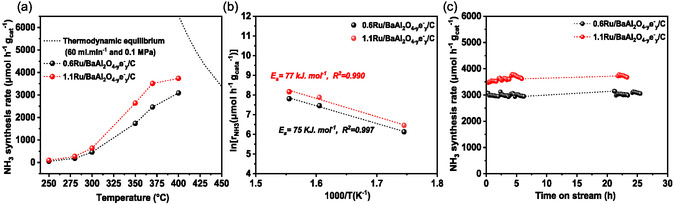
a) Temperature dependence of the NH_3_ production rates over 0.6Ru/BaAl_2_O_4−*y*_e^−^_*y*_/C and 1.1Ru/BaAl_2_O_4−*y*_e^−^_*y*_/C; b) Arrhenius plots in the 573–643K temperature interval; and c) catalyst stability over time. Ammonia synthesis reaction conditions: 0.1 g of catalysts at 400 °C and 1 bar under 60 mL min^−1^ of 75 vol% H_2_ in N_2_ gas flow.

**Figure 7:**
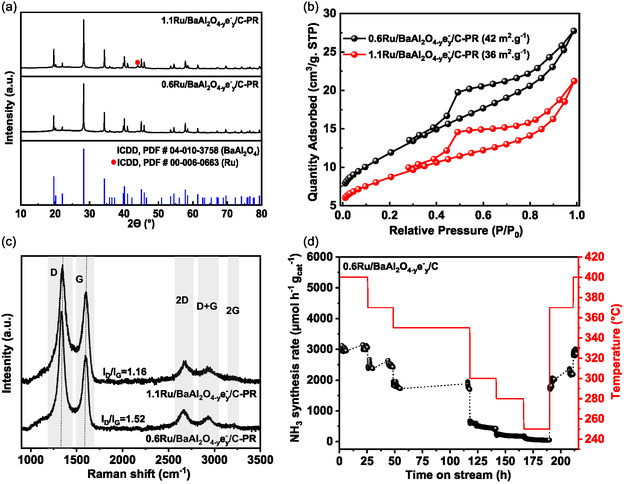
Postreaction characterization after 200h reaction: a) XRD pattern. b) N_2_‐physisorption isotherms. c) Raman spectra and d) catalyst stability over time. Ammonia synthesis reaction conditions: 0.1 g of catalysts at 400 °C and 1 bar under 60 mL min^−1^ of 75 vol% H_2_ in N_2_ gas flow.

The catalytic activity of 0.6Ru/BaAl_2_O_4−*y*_e^−^_*y*_/C and 1.1Ru/BaAl_2_O_4−*y*_e^−^_*y*_/C for ammonia synthesis was measured at ambient pressure under the following conditions: 60 mL min^−1^ of 75 vol% H_2_ in N_2_, WHSV of 36,000 mL g_cat_^−1^ h^−1^, with temperatures ranging from 250 to 400 °C. The catalytic activity was measured without any prior activation. The results are presented in **Figure** **6**,**7**d, Figure S14, Supporting Information, and **Table** **3**.

**Table 3:** Summary of the catalytic activity of BaAl_2_O_4−*y*_e^−^_*y*_/C, 0.6Ru/BaAl_2_O_4−*y*_e^−^_*y*_/C, 1.1Ru/BaAl_2_O_4−*y*_e^−^_*y*_/C for ammonia synthesis under atmospheric pressure.

	Rate [μmol g_cat_^−1^ h^−1^]				Rate [mmol g_Ru_^−1^ h^−1^]				Ea [kJ mol^−1^]
	400 °C	370 °C	350 °C	300 °C	400 °C	370 °C	350 °C	300 °C	
No catalyst	n.m.	–	–	–	–	–	–	–	–
BaAl_2_O_4−*y*_e^−^_*y*_/Ca)	18	–	–	–		–	–	–	–
0.6Ru/BaAl_2_O_4−*y*_e^−^_*y*_/Cb)	3090	2464	1737	460	515	411	290	77	75
1.1Ru/BaAl_2_O_4−*y*_e^−^_*y*_/Cb)	3737	3511	2640	636	340	331	240	58	77

a) mass of material is 0.3 g under 60 mL min^−1^ of 75 vol% H_2_ in N_2_ (Air Products, 99.98%) at 400 °C;

b) Reaction under 60 mL min^−1^ of 75 vol% H_2_ in N_2_ (Air products, 99.98%) with 0.1 g of material, under 1 bar total pressure.

As expected, the BaAl_2_O_4−*y*_e^−^_*y*_/C support, without Ru, displayed, after stabilization under the reaction conditions, a very low ammonia production rate (Table 3; Figure S13, Supporting Information). However, upon Ru introduction, the catalysts namely 0.6Ru/BaAl_2_O_4−*y*_e^−^_*y*_/C, 1.1Ru/BaAl_2_O_4−*y*_e^−^_*y*_/C displayed remarkable catalytic activity even at temperatures below 400 °C (Figure 6a, Table 3). For instance, an ammonia synthesis rate of ≈460 μmol g_cat_^−1^ h^−1^ is measured with 0.6Ru/BaAl_2_O_4−*y*_e^−^_*y*_/C at 300 °C. This is particularly noteworthy, as it demonstrates catalytic performance at significantly lower temperatures compared to some of the most studied traditional catalytic systems such as nitride‐based catalysts Co_3_Mo_3_N, Ni_2_Mo_3_N, and Mo_2_N,^[^ ^59^, ^60^, ^61^, ^62^ ^]^ which typically require operating temperatures of 400 °C or higher to achieve comparable activity. For example, Co_3_Mo_3_N achieves rates of 330–652 μmol h^−1^ g^−1^ at 400 °C and atmospheric pressure. Even when Co_3_Mo_3_N or Mo_2_N are in divided form such as CoMoN/SBA‐15, their performance at such mild conditions remains very limited.^[^ ^63^, ^64^, ^65^ ^]^ Upon increasing the temperature, the catalytic activity increased reaching 3090 μmol g_cat_^−1^ h^−1^. These catalytic activities are far above the value obtained when Ru is supported on the oxide BaAl_2_O_4_ (1377 μmol g_cat_^−^^1^ h^−^^1^ at 400 °C; Table S2, Supporting Information). Theoretically, at our reaction conditions, the maximum ammonia yield at 400 °C is ≈0.4%, which is equivalent to ≈6428 μmol g_cat_^−1^ h^−1^. By comparison, our most active catalyst reaches 3737 μmol g_cat_^−1^ h^−1^ corresponding to 58% of the theoretical maximum highlighting the high potential of these system.

When the catalytic activity is normalized to the Ru weight loading, steady state reaction rate of ≈515 mmol g_Ru_^−1^ h^−1^ is obtained at 400 °C. As it could be awaited considering no significant variation of Ru dispersion, increasing Ru loading in the catalyst results in an increase in catalytic activity (Figure 6a, Table 3). The activation energies (*E*_*a*_) obtained for the reaction with 0.6Ru/BaAl_2_O_4−*y*_e^−^_*y*_/C and 1.1Ru/BaAl_2_O_4−*y*_e^−^_*y*_/C, calculated from the Arrhenius plot, being ≈75 and ≈77 kJ mol^−1^, respectively (Figure 6b). These values are in agreement with *E*_*a*_ previously reported for active electrides, including Ru (2 wt%) /BaAl_2_O_4−*x*_H_*y*_ (65.7 kJ mol^−1^) and Ru (2 wt%)/C12A7:e^−^ (72 kJ mol^−1^).^[^ ^11^, ^34^ ^]^ Such activation energies, in the 40–80 kJ mol^−1^ interval, are directly comparable to those obtained for the most active catalysts reported in the literature, including Ru/BaO_*x*_N_*y*_:e^−^_*z*_(*x* = 0.5), Ru/C12A7:e^−^, Ru/BaScO_2_H, Ru/BaYO_2_H, Ru/BaTiO_2.5_H_0.5_, Ru/Y_5_Si_3_, >Ru/LaScSi, LaRuSi, Ru/BaCeO_3−*x*_N_*y*_H_z_, CoMo/CeO_2_, Li‐MT, Mn_4_N‐BaH, and Re/Ni/Co‐Mo_2_CT_*x*_ (Table S2, Supporting Information).^[^ ^13^, ^14^, ^16^, ^18^, ^19^, ^20^, ^66^, ^67^, ^68^, ^69^, ^70^, ^71^, ^72^ ^]^

In this work, Ru/BaAl_2_O_4−*y*_e^−^_*y*_/C catalysts have demonstrated significantly higher catalytic activity under mild conditions, that surpasses the performances of classical Ru‐based catalysts. Although, the presence of graphitized carbon (≈20 wt%) in BaAl_2_O_4−*y*_e^−^_*y*_/C may raise questions about its potential role in promoting the activity of Ru. However, the performance of Ru/BaAl_2_O_4−*y*_e^−^_*y*_/C surpasses by far that of conventional Ba‐Ru/AC (148 μmol g_cat_^−^^1^ h^−^^1^), Ba‐Ru/graphene (336 μmol g_cat_^−^^1^ h^−^^1^), and K‐Ru/graphite (490 μmol g_cat_^−^^1^ h^−^^1^), Table S2, Supporting Information.^[^ ^16^, ^73^, ^74^ ^]^ Therefore, the catalytic performance of Ru/BaAl_2_O_4−*y*_e^−^_*y*_/C composite cannot be attributed solely to the presence of graphitic support, but rather to the BaAl_2_O_4−*y*_e^−^_*y*_/C component playing a critical role in enhancing the catalytic activity of Ru at lower temperatures. These findings establish Ru/BaAl_2_O_4−*y*_e^−^_*y*_/C as a highly efficient catalyst for ammonia synthesis under mild conditions (*P*_atm_ and <400 °C).

To rule out the influence of inorganic impurities, although present in small quantities in the composites, control experiments were conducted using catalysts with composition similar to the extra‐phases detected in the samples (e.g., AlN, BaO). Reference catalysts such as 2.5Ru/AlN/C, 4Ba‐Ru/AlN/C, exhibited much lower catalytic activity compared to the Ru/BaAl_2_O_4−*y*_e^−^_*y*_/C catalysts (Table S2, Supporting Information) confirming that the superior performance when Ru is supported on the oxy‐electride composite support.

### 2.6 Catalytic Stability of Ru/BaAl_2_O_4−*y*_e^−^_*y*_/C

Stability is a sine‐qua‐non condition for any catalyst to be of practical industrial importance. The catalytic activity of 0.6Ru/BaAl_2_O_4−*y*_e^−^_*y*_/C was measured at 400 °C for 24 h of ammonia synthesis reaction (Figure 6c). The catalytic activity is found stable. The robustness of the catalyst is also confirmed under temperature variation activity measurement (Figure S14, Supporting Information). After the first cycle of catalytic activity is conducted under temperature decrease from 400 to 250 °C, the reaction temperature is increased back to 400 °C and the activity is measured at a comparable value (<5% variation) than initially: initial period, at 3090 μmol g_cat_^−1^ h^−1^, final period at 2900 μmol g_cat_^−1^ h^−1^ (Figure 7d). Similar stability is observed for 1.1Ru/BaAl_2_O_4−*y*_e^−^_*y*_/C catalyst (Figure 6c). Textural and structural characterization was conducted (Figure 7) after reaction. After 215 h of reaction (0.6Ru/BaAl_2_O_4−*y*_e^−^_*y*_/C), XRD pattern remains identical to the initial one (Figure 7a). In the case of 1.1Ru/BaAl_2_O_4−*y*_e^−^_*y*_/C (140 h of reaction), a small diffraction peak at 2*θ* ≈ 44° (Ru metal phase, ICDD, PDF #00‐006‐0663) appears, which may denote small sintering of Ru over this sample.

Furthermore, no noticeable changes in the accessible surface area were observed after the stability test was conducted (Figure 7b). For instance, the changes in the surface area, of 0.6Ru/BaAl_2_O_4−*y*_e^−^_*y*_/C, within the range of the error interval: 42 m^2^ g^−1^ after reaction, versus 47 m^2^ g^−1^ for the as‐prepared catalyst. Additional SEM analysis confirms that the catalysts retained comparable morphology of aggregates after reaction (Figure S15, Supporting Information). Carbon content as well as its nature in the composite after reaction is also evaluated (Raman spectroscopy, Figure 7c, and TGA‐DTA under air; Figure S16, Supporting Information). Raman signature and carbon content do not significantly change after reaction: I_D_/I_G_ ratio is ≈1.52, to be compared to ≈1.23 for 0.6Ru/BaAl_2_O_4−*y*_e^−^_*y*_/C; carbon content is 25.0 wt% after reaction, to be compared to 26.9 wt% for 0.6Ru/BaAl_2_O_4−*y*_e^−^_*y*_/C. All these observations are indicative of a limited modification of either the composite support or the Ru active phase during reaction.

## 3 Discussion

In this work, we present a simple route to produce electride‐based composite, based on a modified Pechini approach, using accessible precursors such as nitrates and carbon source (citric acid and ethylene glycol), that is followed by carbothermal reduction at 1400 °C under nitrogen. The modified Pechini method offers an accessible alternative to classical high‐temperature or vacuum‐based synthesis routes for electrides, thus broadening the practical application range of electride materials.

As shown by XRD, high‐resolution TEM, and Raman analysis, the carbothermal reduction step not only leads to the formation of the BaAl_2_O_4_ structure but also generates a graphitic‐like carbon matrix. Raman spectroscopy confirmed the formation of sp^2^‐rich graphitized carbon with moderate disorder (I_D_/I_G_ ratio ≈1.17). This interplay between the oxide and the carbon leads to the formation of a composite that retains high surface area (≈40 m^2^ g^−^^1^) and satisfying electron density (>5 × 10^21^ cm^−3^) as confirmed by iodometric titration and EPR. Importantly, the composite remains stable under ambient handling, addressing a typical vulnerability of many electride systems, which often degrade upon exposure to air or moisture.

Iodometric titration and EPR analysis confirm the presence of anionic electrons within the composite, which enhance electron mobility and is described as promoting strong electron donation, which is an essential to promote the catalytic activity of Ru especially at temperature below 400 °C.^[^ ^22^, ^31^, ^32^, ^33^ ^]^ Previous work by Hosono et al. demonstrated that BaAl_2_O_4−*x*_H_*y*_ surfaces can exhibit exceptionally low work functions due to the presence of “nucleus‐free” anionic electrons.^[^ ^11^, ^33^ ^]^ These extra electrons when transferred to the supported metal phase (e.g., Ru, Co), result in lowering the energetic barrier for direct dinitrogen dissociation, which is the rate‐determining step in most catalyst for ammonia synthesis.^[^ ^11^, ^33^, ^34^ ^]^ As such, the high catalytic activity observed in our composites stems from the presence of such anionic electrons.

Upon Ru impregnation, TEM analysis revealed uniform Ru dispersion with nanoparticles size ≈1.7–2 nm, which is optimal for maximizing B5‐type site proportion. As expected, since the characterization results confirmed the electride‐like nature of the composite support and appropriate Ru particle size, Ru‐loaded composites achieved remarkable ammonia synthesis rates, reaching >3000 μmol g_cat_^−1^ h^−1^ (0.6 wt% Ru) and >3700 μmol g_cat_^−1^ h^−1^ (1.1 wt% Ru) at 400 °C. These performances far exceed those of traditional Ru‐based catalysts, as can be observed in Table S2, Supporting Information, reporting activities in key studies on ammonia synthesis. Estimated activation energies (≈60 kJ mol^−1^) are comparable to those of state‐of‐the‐art electride‐based catalysts (e.g., Ru/C12A7:e^−^ and Ru/BaAl_2_O_4−*x*_H_*y*_), being significantly lower than those of conventional Ru/carbon catalysts (80–130 kJ mol^−1^).

The stability of the Ru/BaAl_2_O_4−*y*_e^−^_*y*_/C catalysts is confirmed through long‐term testing. After 9 days of operation, the catalyst retained its activity, confirmed by a structural integrity retained for both the composite support and the Ru phase. Overall, 0.6Ru/BaAl_2_O_4−*y*_e^−^_*y*_/C catalysts demonstrate excellent performances for ammonia synthesis under mild conditions, with activity and stability rivaling with those of advanced electride‐based catalysts, while being obtained by a simple modified Pechini method. At higher Ru loading, 1.1Ru/BaAl_2_O_4−*y*_e^−^_*y*_/C, a minor diffraction peak at 2*θ* ≈ 44°, attributed to metallic Ru (ICDD, PDF #00‐006‐0663), was observed after 215 h of reaction suggesting slight particle growth and possible change in size distribution. While the catalytic activity was stable overtime, increasing the Ru does indeed lead to an increase in overall catalytic activity per gram of catalyst. However, this improvement does not scale linearly when normalized to the amount of active phase (Ru). In fact, as presented in Table 3, the activity per gram of Ru actually decreases slightly at higher loadings. This suggests that beyond a certain point, increasing Ru content may not lead to expected results, possibly due to reduced dispersion and decreases in B5‐type active sites density.

## 4 Conclusion

BaAl_2_O_4−*y*_e^−^_*y*_/C composite, prepared by a simple Pechini procedure followed by a carbothermal reduction, is found to be a suitable support for Ru dispersion. The catalyst exhibits high catalytic activity under mild reaction conditions (temperatures below 400 °C and ambient pressure), and demonstrates high stability over time.

Comprehensive characterizations using XRD, N_2_‐physisoprtion, SEM, STEM‐HAADF, iodometric titration, and EPR confirmed textural, structural, and electronic properties of the BaAl_2_O_4−*y*_e^−^_*y*_/C oxy‐electride composites. Catalytic performance evaluation confirmed the high performance of Ru/BaAl_2_O_4−*y*_e^−^_*y*_/significantly outperforming traditional Ru‐based catalysts. Notably, at steady state condition, a rate of >3000 μmol g_cat_^−1^ h^−1^ is achieved with 0.6Ru/BaAl_2_O_4−*y*_e^−^_*y*_/C at 400 °C and 1 bar. This is remarkable considering the minimal of Ru (0.6 wt%) loading, especially when compared to reference studies typically using 1–2.5 wt% Ru. Furthermore, the catalysts developed in this study demonstrated excellent stability, maintaining activity over reaction cycle (215 h, with temperature ranging from 400 to 250 °C) highlighting high stability.

Overall, the complexation‐carbothermal method reported here, owing to its simplicity and the excellent properties of the materials obtained (stability and activity) represents a significant advancement toward the practical application of electride‐based materials in sustainable chemical processes, offering a promising pathway for the development of green ammonia synthesis technologies.

## 5 Experimental Section

5.1

5.1.1

##### Synthesis of Support Materials

The synthesis of BaAl_2_O_4−*y*_e^−^_*y*_/C oxy‐electride composite was performed using a pseudo‐Pechini complexation method, followed by carbothermal reduction. The required amount of aluminum nitrate nonahydrate (17.9932 g) and barium nitrate (6.2044 g) were dissolved in distilled water (*V* = 200 mL). The solution was then added to an EG solution (*V* = 16.2 mL) at 60 °C under stirring. Subsequently, citric acid (molar ratio citric acid to metallic cation of 1:1) was added. The resulting solution was heated to 80 °C for 4 h to form a transparent and stable gel. Excess of water was then evaporated at 200 °C for 6 h resulting in the formation of a xerogel. The resulting solid was crushed and subjected to carbothermal reduction at 1400 °C (4 °C min^−1^, 4 h, N_2_ flow rate of 200 mL min^−1^).

For comparison purposes, BaAl_2_O_4_ oxide was prepared according to the above‐described procedure, followed by a calcination at 1000 °C for 20 h under static air.

##### Preparation of Ru‐Based Catalysts

Ru/BaAl_2_O_4−*y*_e^−^_*y*_/C samples, with different Ru loadings, are prepared by impregnation.^[^ ^75^ ^]^ BaAl_2_O_4−*y*_e^−^_*y*_/C was dispersed in tetrahydrofuran (THF, *V* = 25 mL), at which appropriate amount of Ru_3_(CO)_12_ was added under stirring. The suspension was maintained at 65 °C for 12 h, before drying at 80 °C for 3 h. The solid was heated 250 °C for 2 h under N_2_ (200 mL min^−1^) for carbonyl decomposition.

##### Nomenclature

Catalysts were denoted as *x*‐Ru/BaAl_2_O_4−*y*_e^−^_*y*_/C where *x* refers to Ru loading confirmed by inductively coupled plasma optical emission spectrometry. Postreaction materials are referred to as *x*‐Ru/BaAl_2_O_4−*y*_e^−^_*y*_/C‐PR.

##### Characterization

Powder XRD experiments were conducted on a Bruker X‐ray AXS D8 Advance diffractometer in Bragg‐Brentano geometry configuration. The instrument was equipped with a LynxEye Super Speed detector and Cu Kα anticathode (*λ* = 1.54184 Å). Analysis was conducted in the 5‐80° range, with 0.02° step size and counting time of 1.5 s per step. Crystal phase identification was carried out by comparison with ICDD database using EVA software. Rietveld fitting was conducted using the JANA2006 software.

N_2_ adsorption‐desorption isotherms were measured at 77K on a Micromeritics Tristar II automated gas sorption system. Prior to the analysis, the samples were heated at 120 °C under dynamic vacuum (12 h). The specific surface area (*S*_BET_) was calculated from the linear part of Brunauer–Emmett–Teller (BET) plot (P/P_0_ = 0.03–0.35).

Iodometric titration was carried out over ≈10 mg of sample in aqueous I_2_ solution (1.0 × 10^−3^ m).^[^ ^76^ ^]^ Hydrochloric acid was added to the solution to allow the sample dissolution. Sonication was applied to ensure complete dissolution. Thereafter, the excess of I_2_ (not reduced by reaction with trapped electrons into iodide ions, I^−^) is titrated using a sodium thiosulfate solution (1.0 × 10^−3^ m) in the presence of starch as colored indicator.

Redox properties evaluation was conducted through H_2_‐TPR experiments, performed using an AutochemII analyzer (Micromeritics). Before measurement, sample (50 mg) was heat treated under argon flow (50 mL min^−1^) at 120 °C for 2 h (10 °C min^−1^) to remove any weakly adsorbed surface species. After cooling down to RT, the sample was flowed under 5 vol% H_2_ in Ar (50 mL min^−1^) from 25 to 1000 °C (5 °C min^−1^, isothermal time at 1000 °C = 30 min). H_2_ concentration is on‐line monitored on a thermal conductivity detector (TCD).

TGA‐DTA experiments were conducted on a STARe System TGA/DSC 3+ from Mettler Toledo. Analyses were conducted under air flow (80 mL min^−1^) in the 25–1000 °C interval (temperature rate 5 °C min^−1^).

Raman spectroscopy spectra were recorded at RT on a XploRA plus spectrometer (Horiba Scientific), equipped with CCD detector cooled through Peltier effect. Samples were subjected to 532 nm laser excitation to acquire spectral data from 200 to 4000 cm^−1^. Illumination was conducted for 60 sec (acquisition time), utilizing a 50x objective lens and a grating of 1600 lines mm^−1^. The LABSPEC software was used for acquisition and data processing. Spectra were decomposed using Lorentzian function (900–1900 cm^−1^), or pseudo‐Voigt function (2400–3300 cm^−1^). Carbon signals in the 900–1900 cm^−1^ interval were fitted with D*, D, D″, and G bands. Carbon signals in the 2400–3300 cm^−1^ interval were fitted with 2D, D+G, and 2G bands.^[^ ^37^, ^77^, ^78^ ^]^

SEM analysis was conducted on a JEOL JSM‐7800F LV microscope, employing a Lower Electron Detector alongside a retractable Robinson‐type backscatter detector (BED‐C) for Z‐contrast imaging. The elemental composition distribution within the samples was discerned utilizing an Oxford Instruments EDS. The analytical parameters comprised a 5 kV accelerating voltage, and a 10.0 mm working distance.

TEM observation was conducted on a TITAN Themis 300 S/TEM, which provides a high‐brightness Schottky field emission source, a monochromator, and a probe aberration corrector. This setup provided an energy resolution of roughly 150 meV and a spatial resolution of around 70 pm. The microscope included annular dark field detectors and a super‐X detector system with four silicon drift detectors for EDS. Experiments were conducted at 300 kV with a semiconvergence angle of ≈20 mrad, a probe size of ≈500 pm, and a probe current of 60–100 pA. HAADF imaging used collection angles of 50–200 mrad. EDS mapping was performed in spectrum imaging mode with a pixel dwell time of ≈15 μs and for total acquisition times of 15–20 min. Sample powder was deposited on Lacey carbon grids.

XPS spectra were recorded on a Kratos Analytical AXIS Ultra DLD spectrometer employing a monochromatic Al Kα X‐ray radiation (1486.6 eV), with an electron analyzer operating in a fixed pass energy of 20 eV. All binding energies (BE) were referenced to the carbon peak corresponding to C—C bonding in the C 1s core level at 284.8 eV. to mimic the catalytic reaction conditions, a pretreatment chamber was used, prior to analysis, at 400 °C for 2 h under a flow of 75 vol% H_2_ in N_2_.

##### Catalytic Activity in Ammonia Synthesis

Reaction was conducted on a fixed‐bed tubular reactor (Figure S17). 100 mg of powder catalyst was placed in a quartz reactor between quartz‐wool plugs. The reaction was conducted under a flow of 75 vol% H_2_/N_2_ (Air Products, 99.98%), at a WHSV of 36,000 mL g^−1^ h^−1^ for temperature ranging from 250 and 400 °C under atmospheric pressure. Reaction was performed without any prior pre‐treatment for catalyst activation. Ammonia production rate was calculated from the decrease of conductivity of a 200 mL 0.0018 m H_2_SO_4_ solution through which the reactor effluent flow was flowed. The ammonia synthesis reaction rate was determined using the following equation

(1) $$r_{\text{NH}3}\frac{\Delta C\times n_{{\text{NH}}_{3}}\times 60}{m\times 10^{-6}\times t}$$

where *r*_NH3_ is the reaction rate expressed in μmol h^−1^ g^−1^; Δ*C* is the average change in conductivity per minute during the given time range; *n*_NH3_ represents the number of moles of ammonia corresponding to a decrease of 1 μS cm^−1^; m is the mass of the catalyst (in grams); *t* is the average time of the reaction (in minutes); and the constants (60 and 10^−6^) are included to ensure proper unit conversions, yielding the reaction rate in the desired units (μmol h^−1^ g^−1^).

The reaction rate is expressed in μmol g_cat_^−1^ h^−1^ or mmol g_cat_^−1^ h^−1^ where g_cat_ refers to the total mass of the catalyst, including both the support and the active phase. For a more comprehensive assessment, the reaction rate was also normalized to the mass of the active metal (ruthenium), and expressed in mmol g_Ru_^−1^ h^−1^.

## Conflict of Interest

The authors declare no conflict of interest.

## Supporting information

Supplementary Material
