# Supplementary material for: Simplified Preparation of BaAl2O4−y e− y /C Oxy‐Electrides Using Pechini Approach for Ammonia Synthesis
Source: ChemSusChem. 2025 Jun 13;18(15):e202500682. doi: 10.1002/cssc.202500682 (PMC12302307; doi:10.1002/cssc.202500682)
Supplement: Supplementary file 1 — Supplementary Material [file CSSC-18-e202500682-s001.pdf]

Supporting Information

Simplified Preparation of $BaAl_2O_{4-y}e^-_y$/C Oxy-Electrides using Pechini Approach for Ammonia Synthesis.

Aissam Addou[1,2], Amanda Sfeir[2], Maya Marinova[3], Hervé Vezin[4], Jean-Philippe Dacquin[2], Sébastien Royer[5]*, Said Laassiri[1]**

[1] Chemical & Biochemical Sciences, Green process Engineering (CBS), Mohammed VI polytechnic university, UM6P, 43150, Benguerir, Morocco.

[2] Université de Lille, CNRS, ENSCL, Centrale Lille, Univ. Artois, UMR 8181-UCCS-Unité de Catalyse et de Chimie du Solide, F-59000 Lille, France.

[3] Université de Lille, CNRS, INRA, Centrale Lille, Université Artois, FR 2638–IMEC–Institut Michel-Eugène Chevreul, 59000 Lille, France.

[4] Laboratoire de Spectroscopie pour Les Interactions La Réactivité et L'Environnement Université de Lille, UMRCNRS 8516-LASIRE, 59000 Lille, France

[5] Université du Littoral Côte d'Opale, UCEIV UR 4492, MREI-2, 59140 Dunkerque, France.

* Corresponding authors: sebastien.royer@eilco.univ-littoral.fr, jean -philippe.dacquin@univ-lille.fr, said.laassiri@um6p.ma

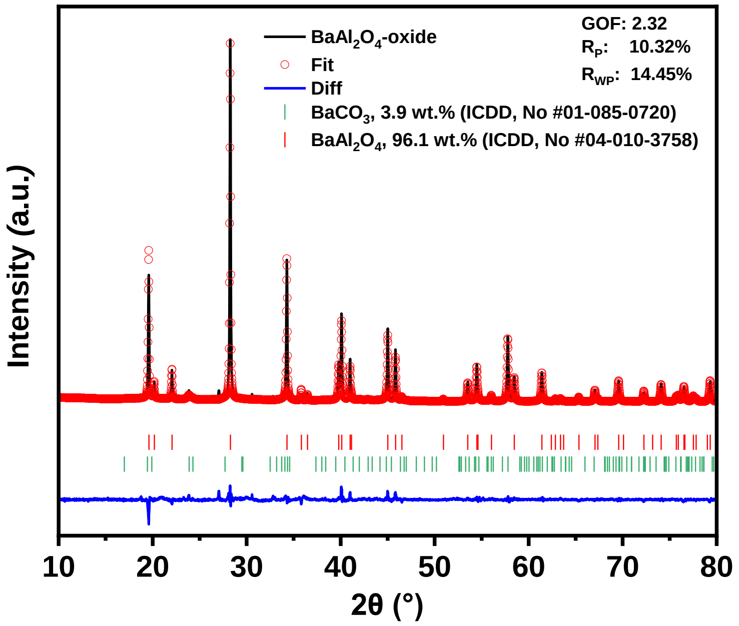

Figure S1. XRD pattern for $BaAl_2O_4$ (calcinated at 1000 °C).

Table S1. Lattice parameters obtained from Rietveld refinement of XRD from sample powders.

Sample	**a[Å]**	**b[Å]**	**c[Å]**	Cell volume **[Å^3]**
$BaAl_2O_4$ oxide	5.2260	5.2260	8.7966	208.1
$BaAl_2O_{4-y}e^-_y$/C	5.2254	5.2254	8.7956	208.0

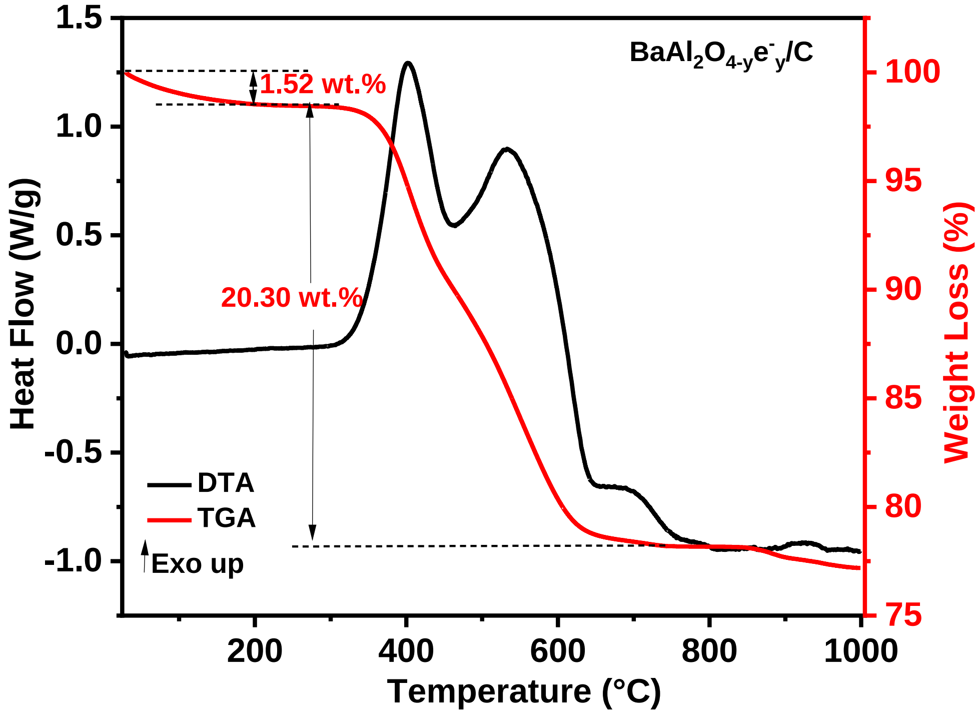

Figure S2. TGA/DTA profiles of the $BaAl_2O_{4-y}e^-_y/C$ when heated under air flow (80 mL min^{-1}, temperature increase rate 5 °C min^{-1}).

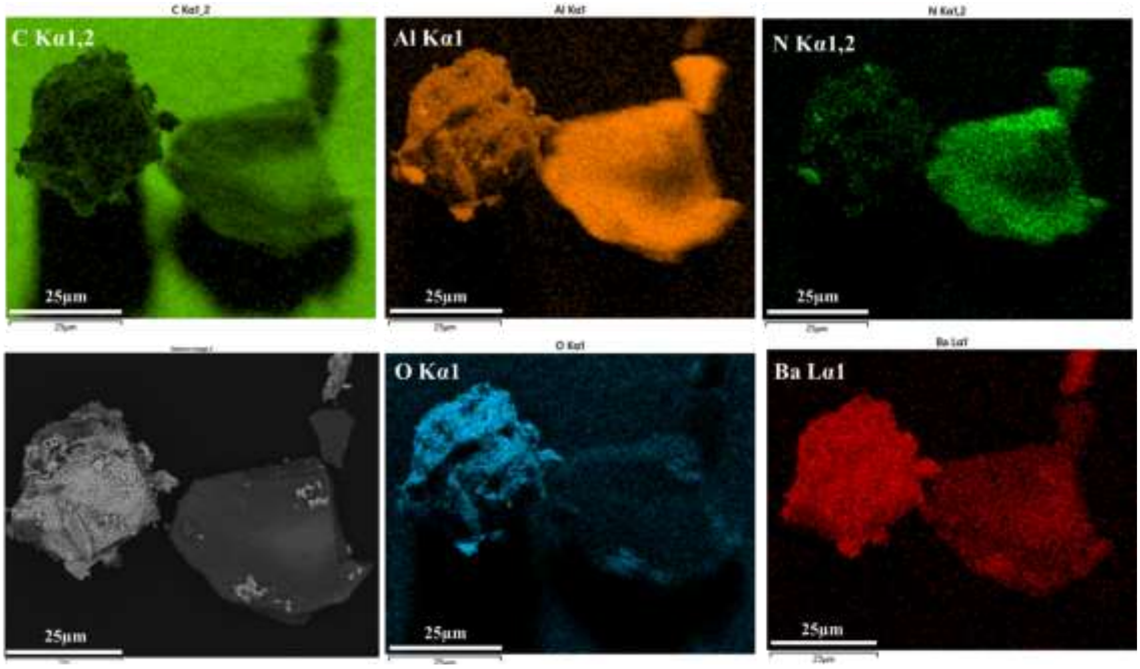

Figure S3. SEM images collected using Robinson-type backscatter detector (BED-C) mode and elemental mapping obtained by EDS spectroscopy for $BaAl_2O_{4-y}e^-_y/C$.

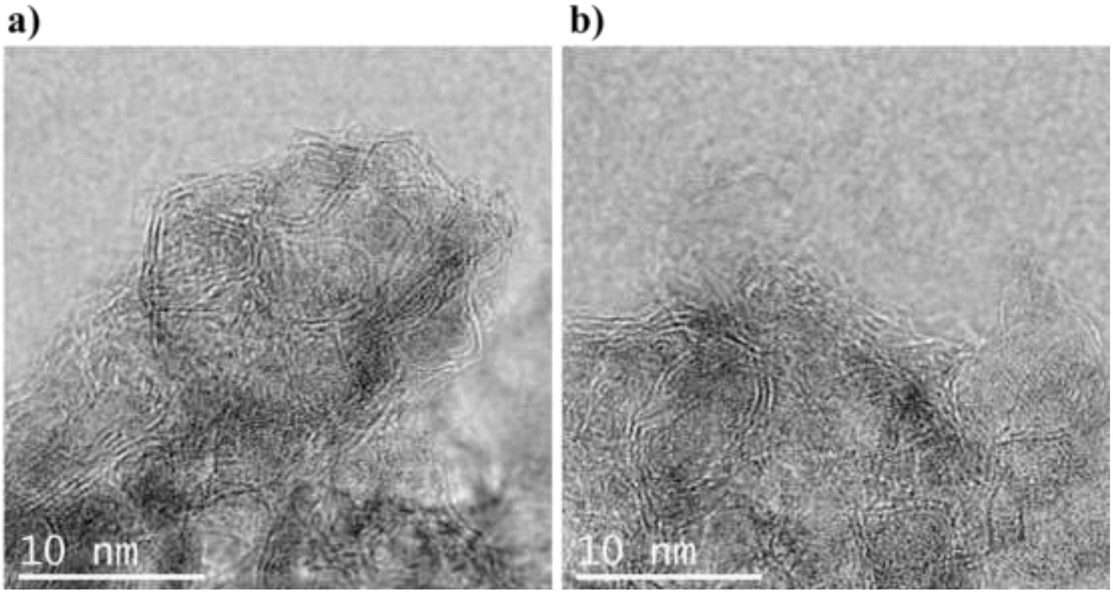

Figure S4. a, b) BF-STEM image showing the sheet-like structures of graphitized carbon in $BaAl_2O_{4-y}e^-{}_y/C$.

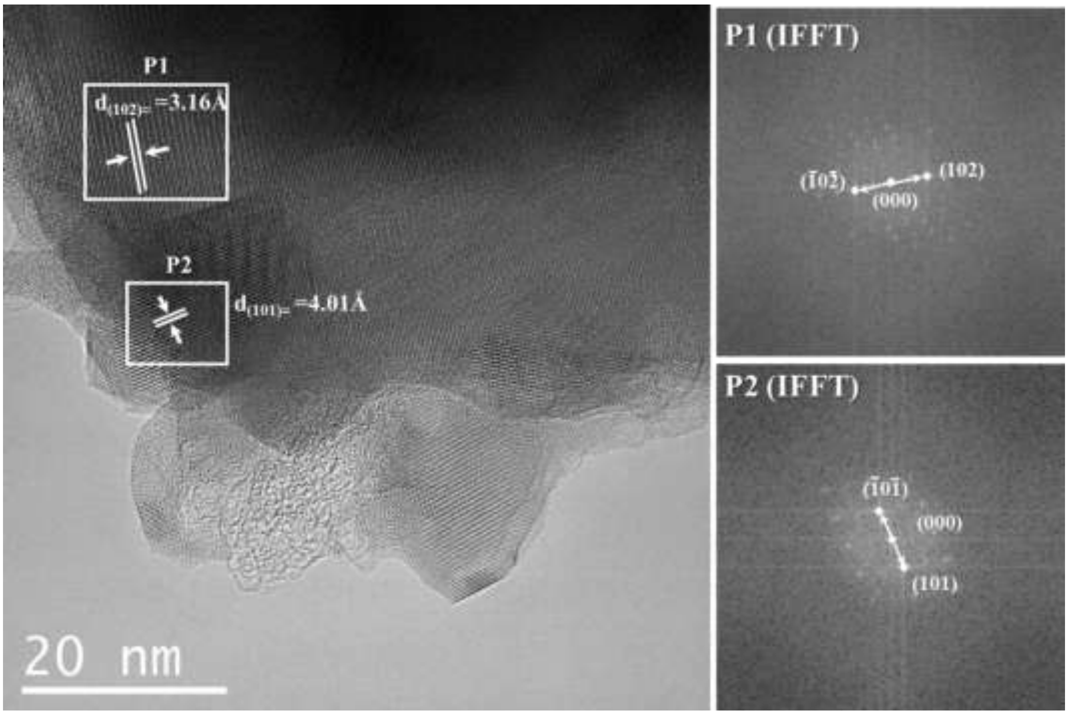

Figure S5. High TEM resolution images of $BaAl_2O_{4-y}e^-{}_y/C$.

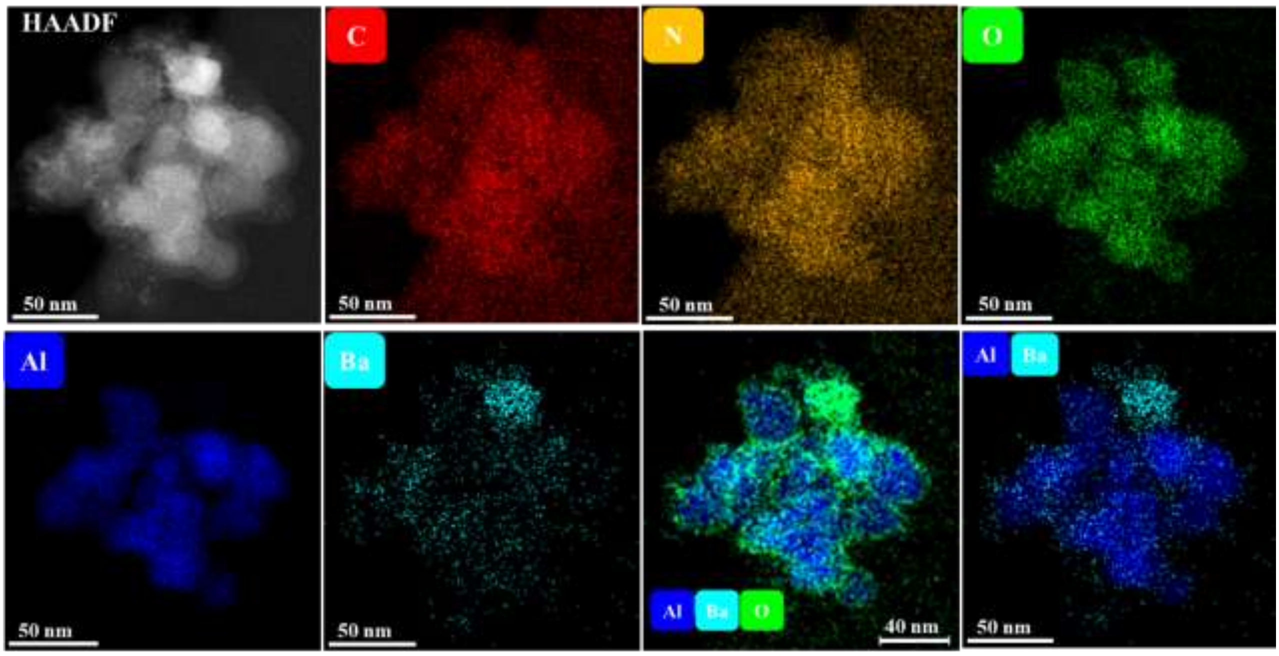

Figure S6. HAADF-STEM coupled EDS elemental mapping of $BaAl_2O_{4-y}e^{-}_{y}$/C.

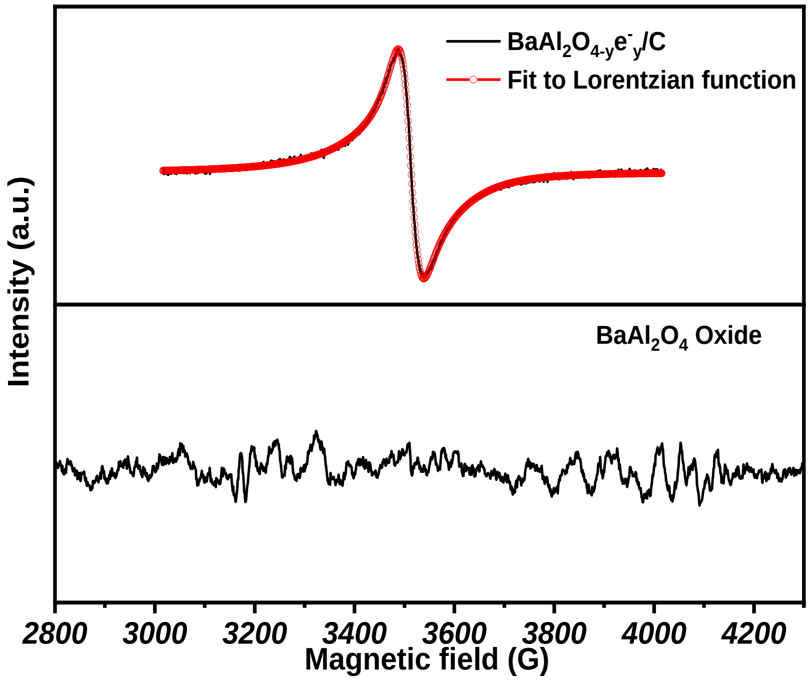

Figure S7. EPR spectrum of $BaAl_2O_{4-y}e^-_y$/C and $BaAl_2O_4$ oxide.

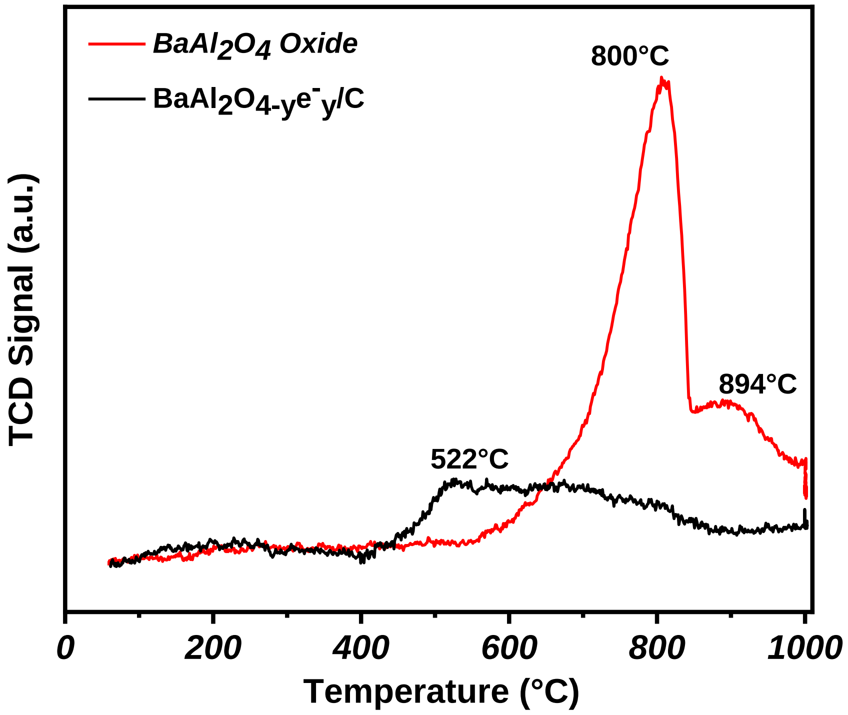

Figure S8. H_2-TPR profile of $BaAl_2O_{4-y}e^-_y$/C and $BaAl_2O_4$ oxide.

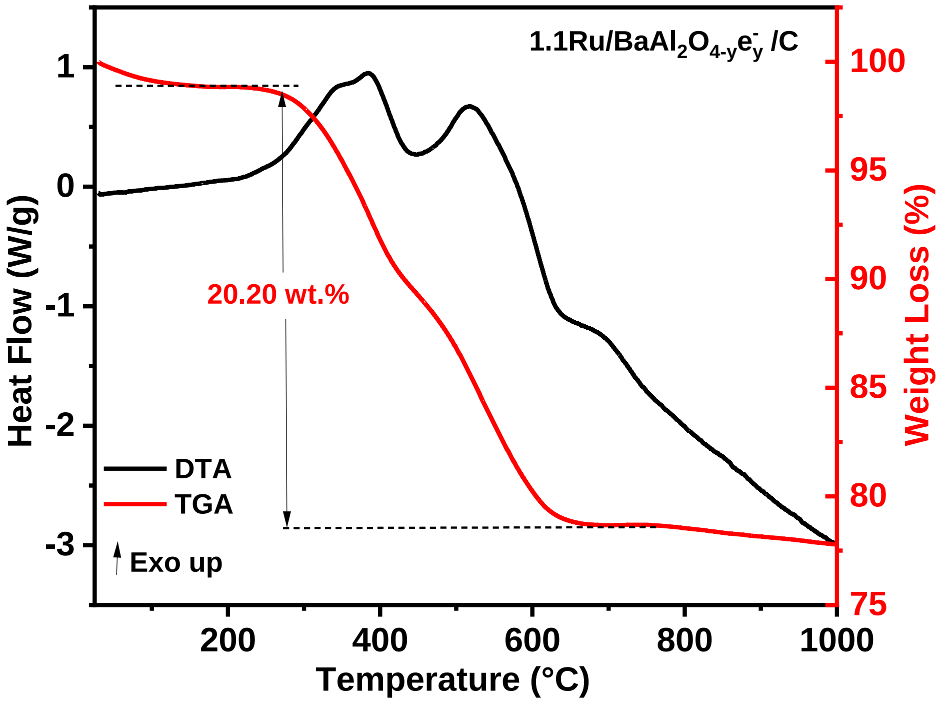

Figure S9. TGA/DTA profile of the 1.1Ru/$BaAl_2O_{4-y}e^-_y$/C when heated under air flow (80 mL min^{-1}, temperature increase rate 5 °C min^{-1}).

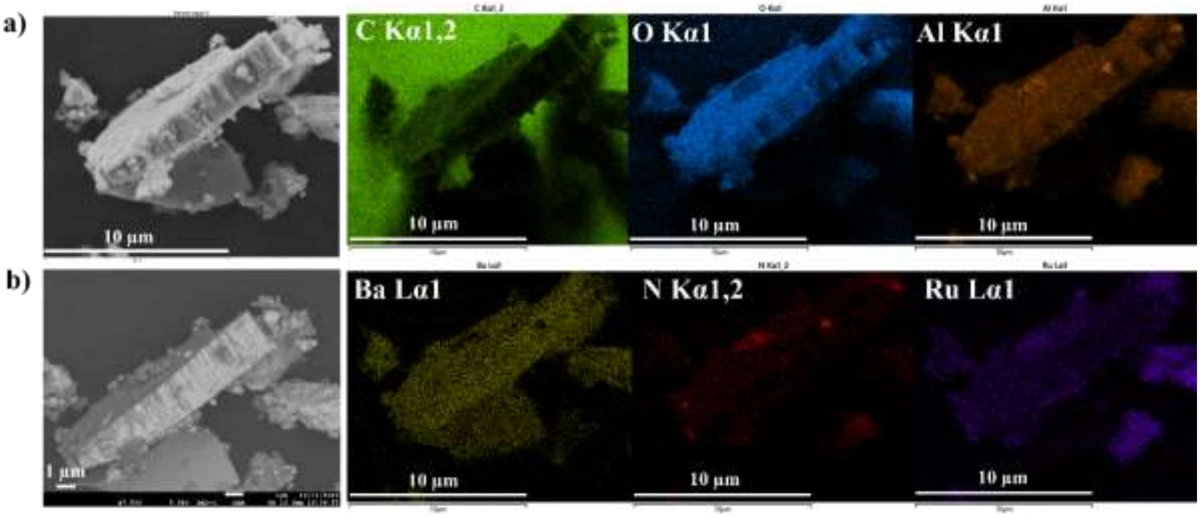

Figure S10. a) SEM micrographs in BDE-C mode; b) SEM using LED mode coupled EDS elemental mapping of 0.6Ru/$BaAl_2O_{4-y}e^-_y$/C.

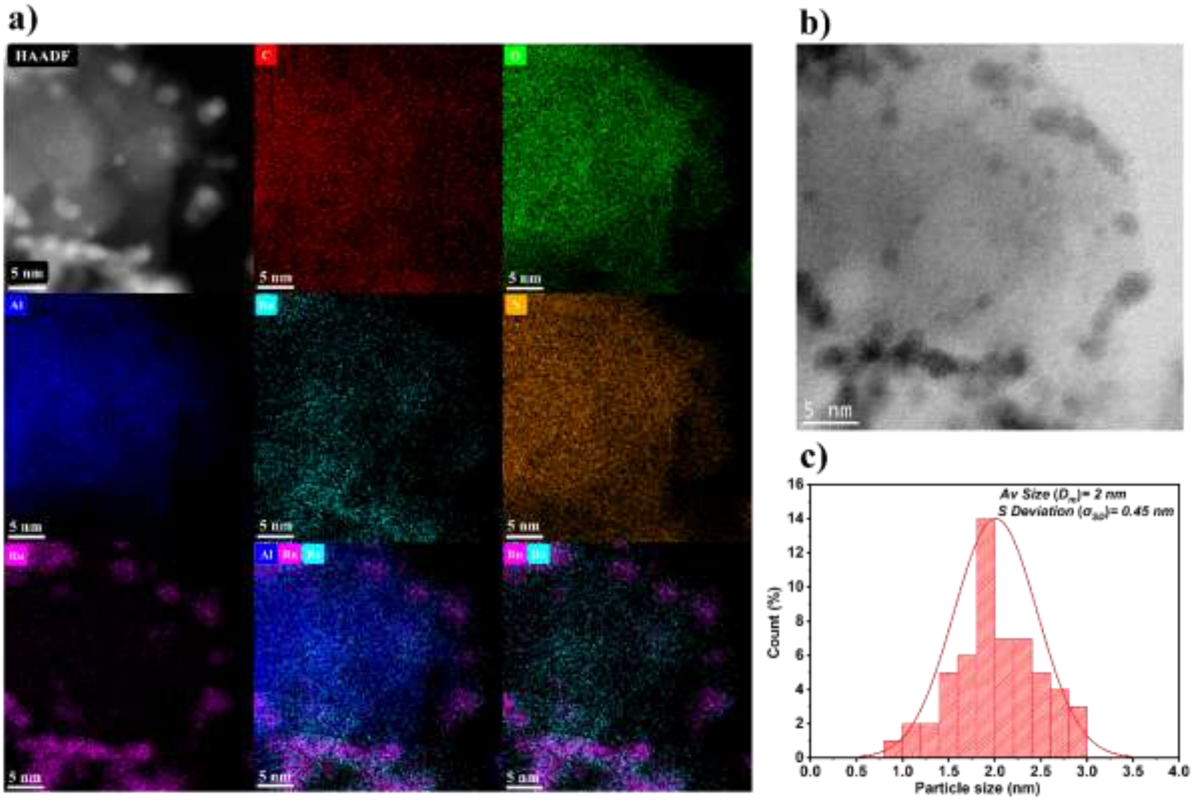

Figure S11. a) HAADF-STEM image coupled with EDS elemental mapping, c) STEM image, and c) corresponding Ru particle size statistical analysis for 1.1Ru/$BaAl_2O_{4-y}e^-_y$/C catalyst.

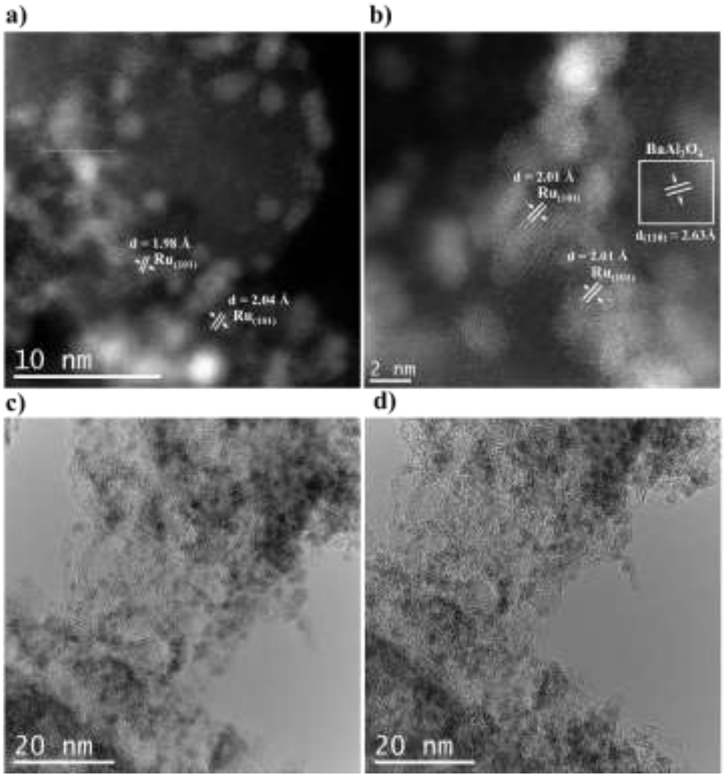

Figure S12. STEM-HAADF images of a) $0.6Ru/BaAl_2O_{4-y}e^-_y$ /C; b) $1.1Ru/BaAl_2O_{4-y}e^-_y$ /C as prepared catalysts. c, d) HR-TEM of $0.6Ru/BaAl_2O_{4-y}e^-_y$ /C as prepared catalyst.

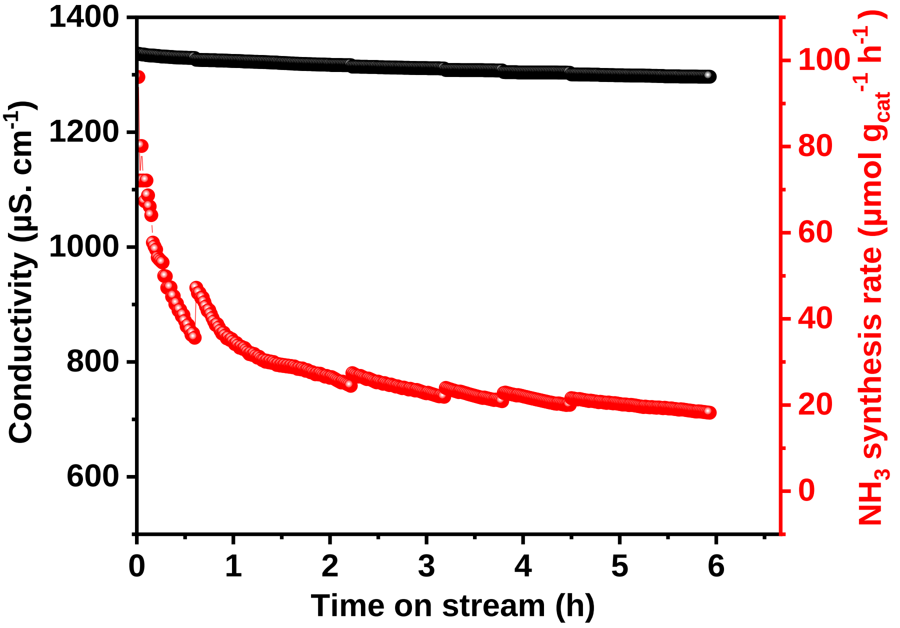

Figure S13. Reaction conductivity and catalytic activity evolution during ammonia synthesis over the $BaAl_2O_{4-y}e^-_y$/C. Conditions of ammonia synthesis reaction: 300 mg of material under a 75% H_2/N_2 flow rate of 60 mL min^{-1} at 400 °C and 1 bar.

Table S2. Catalytic performance of Ru from this work, compared to Ru-based catalysts from the literature.

	Catalyst	Ru[a] [wt%]	SBET[b] [m^2 g^{-1}]	d[c] [nm]	T [°C]	P [MPa]	WHSV [mL g^{-1} h^{-1}]	Rate [mmol g^{-1} h^{-1}]	Rate [mmol g_{Ru}^{-1} h^{-1}]	Ea[d] [kJ mol^{-1}]	Ref.
Ru/inorganic electride	**$BaAl_2O_{4-y}e^-_y/C$**	**-**	**41**	**-**	**400**	**0.1**	**36000**	**0.016**	**0.016**	**-**	**This work**
	**$Ru/BaAl_2O_{4-y}e^-_y/C$**	**0.6**	**47**	**1.7**	**400**	**0.1**	**36000**	**3.090**	**515**	**75**	**This work**
	**$Ru/BaAl_2O_{4-y}e^-_y/C$**	**1.1**	**27**	**2.0**	**400**	**0.1**	**36000**	**3.737**	**340**	**77**	**This work**
	$Ru/BaO_xN_y:e^-_z$ (x=0.5)	1.8	0.7	122	400	0.1	36000	2.050	113.89	62	[S1]
	LaRuSi	-	1	-	400	0.1	36000	1.760	-	40.4	[S2]
	Ru/Y_5Si_3	2	1~2	-	400	0.1	18000	0.8	40	48.0	[S3]
	Ru/CeScGe	1.6	~1	5.6	400	0.1	36000	0.792	49.50	98	[S4]
	Ru/CeScSi	1.7	~1	11.5	400	0.1	36000	2.40	141.17	87	[S4]
	Ru/SP-C12A7:e-	4	1~2	68.5	400	0.1	18000	2.122	53.05	56.0	[S5]
	Ru/SP-C12A7:e-	1.2	1~2	41.3	400	0.1	18000	2.757	229.75	49.1	[S5]
	Ru/HT-C12A7:e-	2	20	9	400	0.1	18000	2.290	114.5	53	[S6]
	Ru(CO)/HT-C12A7	2	35	2	400	0.1	36000	3.010	150.5	-	[S7]
	Ru(Cl)/HT-C12A7	2	34	2	400	0.1	36000	3.053	152.65	-	[S7]
	$Ru/BaYO_2H$	1.8	2.2	8.6	400	0.1	36000	2.77	154	54.2	[S8]
	$Ru/BaScO_2H$	1.7	4.4	6.3	400	0.1	36000	4.09	240.58	48.9	[S8]
Ru/carbon-based support	Ru/MCP-1100°C-N_2	10	545	2.5	510	0.1	6600	1.334	13.34	-	[S9]
	K-Ru/graphite	10	-	-	400	0.1	-	0.490	4.9	-	[S10]
	Ba-Ru/graphene	1.4	168	8.42	400	0.1	36000	0.336	24	-	[S11]
	K-Ru-Co@N-C	1.23		2.0	400	1	12000	0.494	40.20	-	[S12]
	Ba-Ru/AC	1	310	5.3	400	0.1	18000	0.148	14.8	88.8	[S5]
	**Ba-Ru/AlN/C**	**2.5** [e]	**533**	**-**	**400**	**0.1**	**12000**	**0.434**	**17.35**	**-**	**This work**
	**Ru/AlN/C**	**2.5** [e]	**494**	**-**	**400**	**0.1**	**12000**	**0.097**	**3.88**	**-**	**This work**
Ru/oxides	Ru/BaOx	1.8	0.8	231	400	0.1	36000	0.05	2.78	-	[S1]
	Ru/CaO	1.5	3	27.2	400	0.1	18000	0.158	5.267	120.1	[S5]
	Ru/ /γ-Al_2O_3	6	170	10.6	400	0.1	18000	0.051	0.85	64.6	[S5]
	**Ru/Al_2O_3**	**5**[e]	**~1**	**-**	**400**	**0.1**	**12000**	**0.05**	**1**	**-**	**This work**
	**$Ru/BaAl_2O_4$**	**2.5** [e]	**~1**		**400**	**0.1**	**12000**	**1.377**	**55.07**	**81.42**	**This work**

[a] Determined by ICP measurement; [b] Measured by nitrogen adsorption-desorption analysis; [c] Particle size was calculated on the basis of STEM micrograph using ImageJ software; [d] Activation energy for ammonia synthesis over catalysts at 0.10 MPa; [e] Nominal Ru loading.

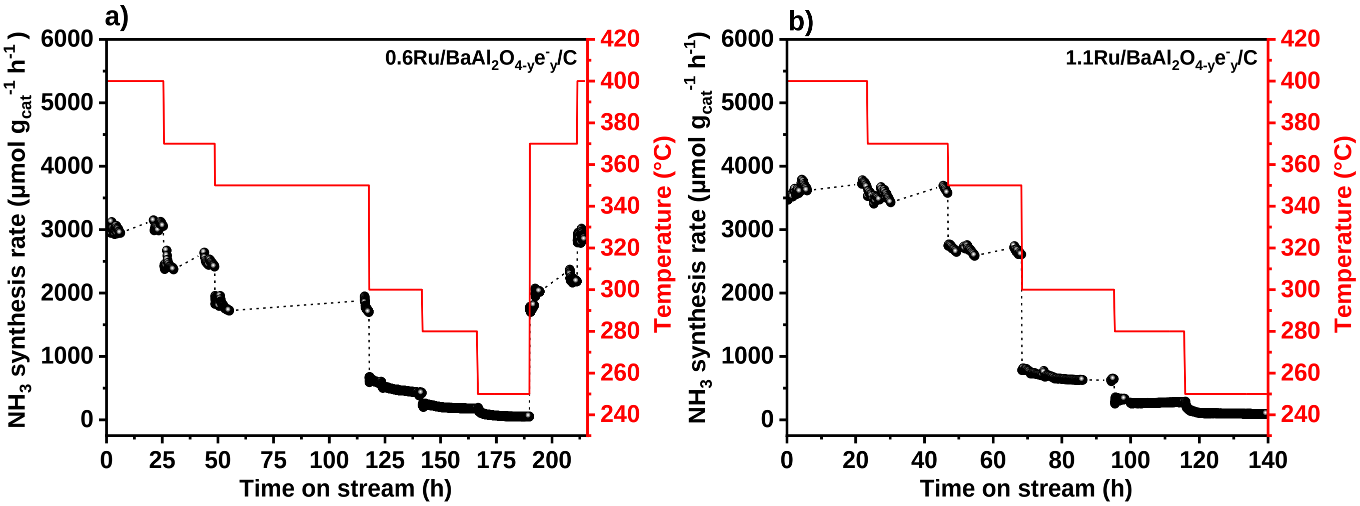

Figure S14. NH_3 production rate over a) 0.6Ru/BaAl$_2$O$_{4-y}$e^-_y/C and b) 1.1Ru/BaAl$_2$O$_{4-y}$e^-_y/C at 0.1MPa at temperature from 400 °C to 250 °C.

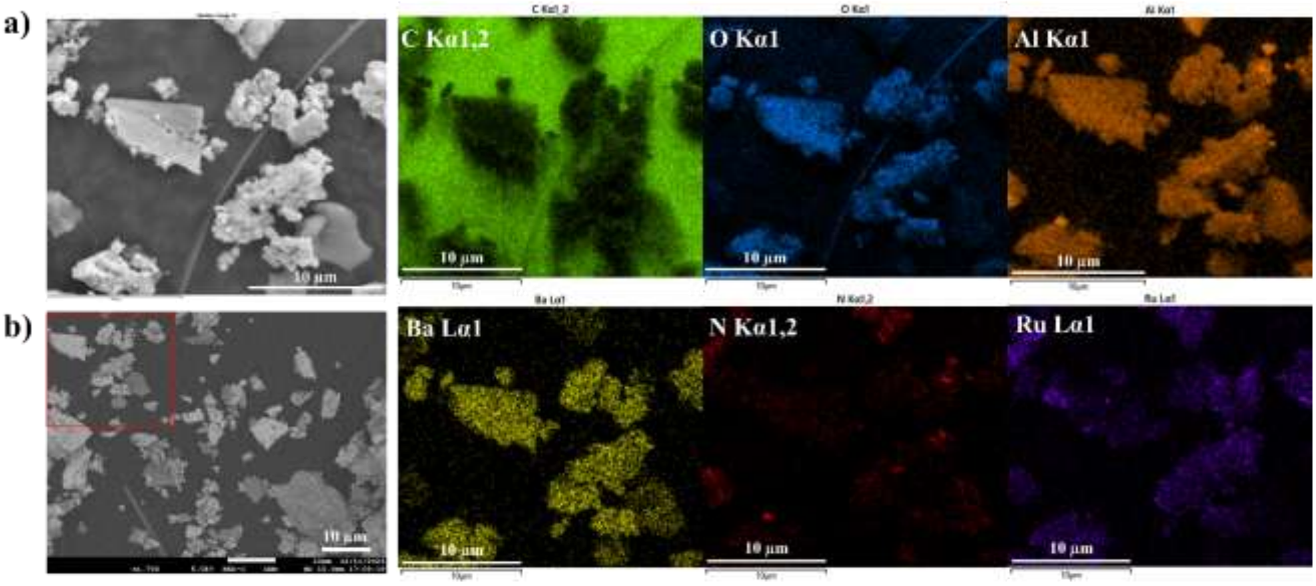

Figure S15. a) SEM using LED mode coupled with EDS elemental mapping; b) SEM using BDE-C mode of 0.6Ru/BaAl$_2$O$_{4-y}$e-$_y$/C-PR.

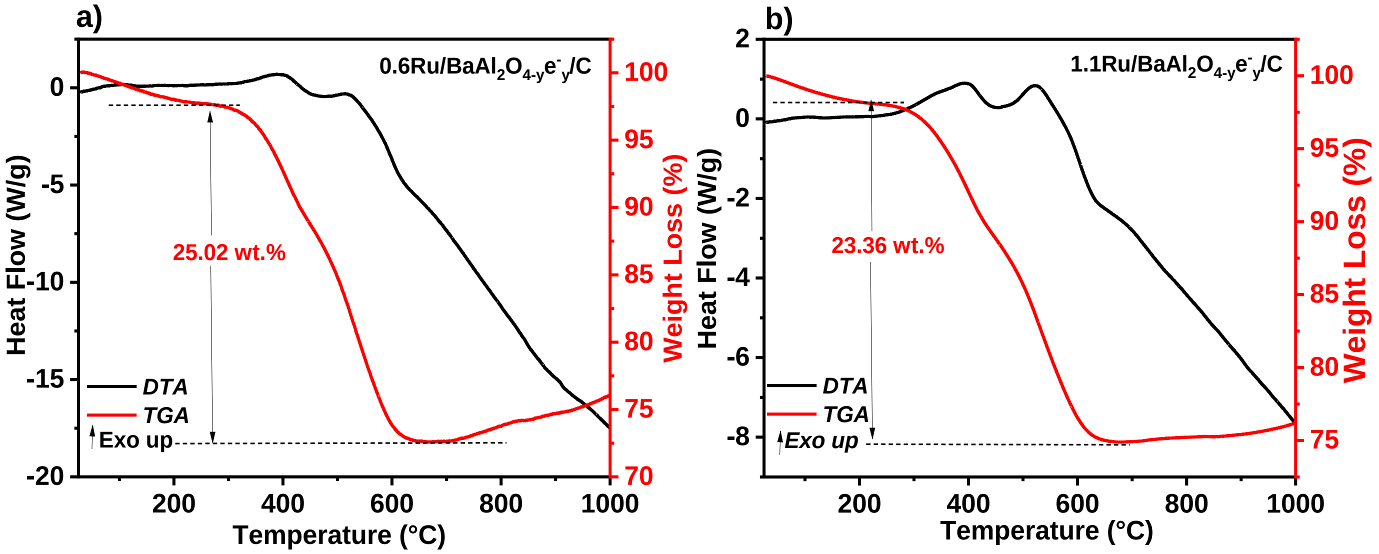

Figure S16. TGA/DTA profiles conducted under air of a) $0.6Ru/BaAl_2O_{4-y}e^-_y/C$-PR and b)$1.1Ru/BaAl_2O_{4-y}e^-_y/C$-PR.

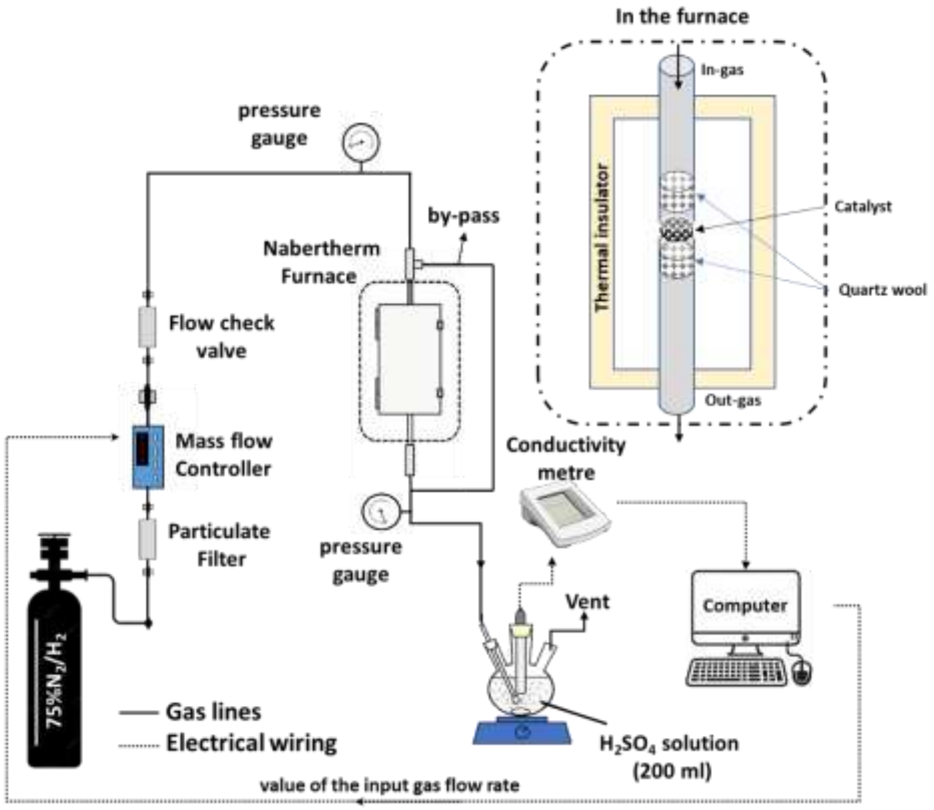

Figure S17. Flowchart of the reaction setup for ammonia synthesis.

References

[S1] J. Li, Y. Jiang, Z. Zhang, M. Tsuji, M. Miyazaki, M. Kitano, H. Hosono, *Adv. Energy Mater*. **2023**, 13, 2302424.

[S2] J. Wu, J. Li, Y. Gong, M. Kitano, T. Inoshita, H. Hosono, *Angew. Chem. Int. Ed*. **2019**, 131, 835.

[S3] Y. Lu, J. Li, T. Tada, Y. Toda, S. Ueda, T. Yokoyama, M. Kitano, H. Hosono, *J. Am. Chem. Soc*. **2016**, 138, 3970.

[S4] C. Croisé, K. Alabd, S. Tencé, E. Gaudin, A. Villesuzanne, X. Courtois, N. Bion, F. Can, *ChemCatChem* **2024**, 16, e202400403.

[S5] M. Kitano, Y. Inoue, Y. Yamazaki, F. Hayashi, S. Kanbara, S. Matsuishi, T. Yokoyama, S. W. Kim, M. Hara, H. Hosono, *Nat. Chem.* **2012**, 4, 934.

[S6] Y. Inoue, M. Kitano, S. Kim, T. Yokoyama, M. Hara, H. Hosono, *ACS Catal.* **2014**, 4, 574.

[S7] J. Li, M. Kitano, T. Ye, M. Sasase, T. Yokoyama, *ChemCatChem* **2017**, 9, 3078.

[S8] S. Sato, M. Miyazaki, S. Matsuishi, H. Hosono, M. Kitano, *Adv. Energy Mater.* **2024**, 14, 2402353.

[S9] S. Y. Chen, L. Y. Wang, K. C. Chen, C. H. Yeh, W. C. Hsiao, H. Y. Chen, M. Nishi, M. Keller, C. L. Chang, C. N. Liao, T. Mochizuki, H. Y. T. Chen, H. H. Chou, C. M. Yang, *Appl. Catal. B: Environ.* **2024**, 346, 123725.

[S10] H. Chen, J. Lin, Y. Cai, X. Wang, J. Yi, J. Wang, G. Wei, Y. Lin, D. Liao, *Appl. Surf. Sci.* **2001**, 180, 328.

[S11] J. Zhao, J. Zhou, M. Yuan, Z. You, *Catal. Lett.* **2017**, 147, 1363.

[S12] J. Yang, D. He, W. Chen, W. Zhu, H. Zhang, S. Ren, X. Wang, Q. Yang, Y. Wu, Y. Li, *ACS Appl. Mater. Interfaces.* **2017**, 9, 39450.
